# Spatial Proteomics Reveals Distinct Protein Patterns in Cortical Migration Disorders Caused by LIN28A Overexpression and WNT Activation

**DOI:** 10.1016/j.mcpro.2025.101037

**Published:** 2025-07-16

**Authors:** Jelena Navolić, Sara Hawass, Manuela Moritz, Jan Hahn, Maximilian Middelkamp, Antonia Gocke, Matthias Dottermusch, Yannis Schumann, Lisa Ruck, Christoph Krisp, Shweta Godbole, Piotr Sumislawski, Nele Köppen, Elisabetta Gargioni, Hartmut Schlüter, Julia E. Neumann

**Affiliations:** 1 Center for Molecular Neurobiology Hamburg (ZMNH), University Medical Center Hamburg-Eppendorf, Hamburg, Germany; 2 Center for Diagnostics: Section Mass Spectrometry and Proteomics, University Medical Center Hamburg-Eppendorf, Hamburg, Germany; 3 Mildred Scheel Cancer Career Center HaTriCS4, University Medical Center Hamburg-Eppendorf, Hamburg, Germany; 4 Institute of Neuropathology, University Medical Center Hamburg-Eppendorf, Hamburg, Germany; 5 Department of Neurosurgery, University Medical Center Hamburg-Eppendorf, Hamburg, Germany; 6 Barts Cancer Institute, Queen Mary University of London, John Vane Science Centre, London, United Kingdom; 7 Chair for High Performance Computing, Helmut Schmidt University, Hamburg, Germany; 8 Department of Neurosurgery, Faculty of Medicine and University Hospital Carl Gustav Carus, Technische Universität Dresden, Dresden, Germany; 9 Laboratory of Radiobiology & Experimental Radiation Oncology, University Medical Center Hamburg-Eppendorf, Hamburg, Germany; 10 Department of Radiotherapy and Radio-Oncology, University Medical Center Hamburg-Eppendorf, Hamburg, Germany

**Keywords:** spatial proteomics, NIRL, LIN28A, CTNNB1, ETMR, lissencephaly

## Abstract

Developmental signaling pathways act in stage and tissue dependent relation and misactivation can drive tumor formation. The RNA-binding protein LIN28A binds to mRNA and miRNA and thereby affects the protein turnover and maintains stemness. LIN28A is overexpressed in embryonal brain tumors which show low correlation between transcriptome and proteome signatures. Additionally, stabilizing *CTNNB1* mutations activating the WNT pathway have been reported in brain tumors with LIN28A overexpression. The aim of this study was to coactivate these oncogenic proteins during embryonal brain development and investigate the histomorphology of the cerebral cortex in relation to proteome levels with spatial resolution using the nanosecond infrared laser system for nano-volume sampling. The combination of both oncogenic factors *in vivo* did not lead to brain tumour formation during embryonal development but resulted in disturbed lamination and impaired cell migration in the murine cerebral cortex. Spatially resolved proteome analysis of the cortices revealed unique layer signatures across ablated layers. Moreover, the extracellular matrix receptors RPSA and ITGB1 were spatially disturbed comparing the mouse models and accompanied by a porous pial border and overmigration of neural cells. Cajal–Retzius cells were misplaced in deeper cortex regions without affecting general REELIN levels. Additionally, the glycosylated levels of α-dystroglycan were reduced. Taken together, the interplay of LIN28A and CTNNB1 resulted in a cortical migration disorder showing histomorphological and molecular similarities to human cobblestone lissencephaly (type 2) disorder. This highlights novel implications of the oncogene LIN28A in extracellular matrix integrity.

## Highlights

• Nano-volume sampling of mouse brain cortices for spatial proteomics.
• The oncogene LIN28A affects extracellular matrix integrity in the brain.
• LIN28A and CTNNB1 lead to a cobblestone lissencephaly type 2-like phenotype.

## Graphical Abstract

**Figure:**
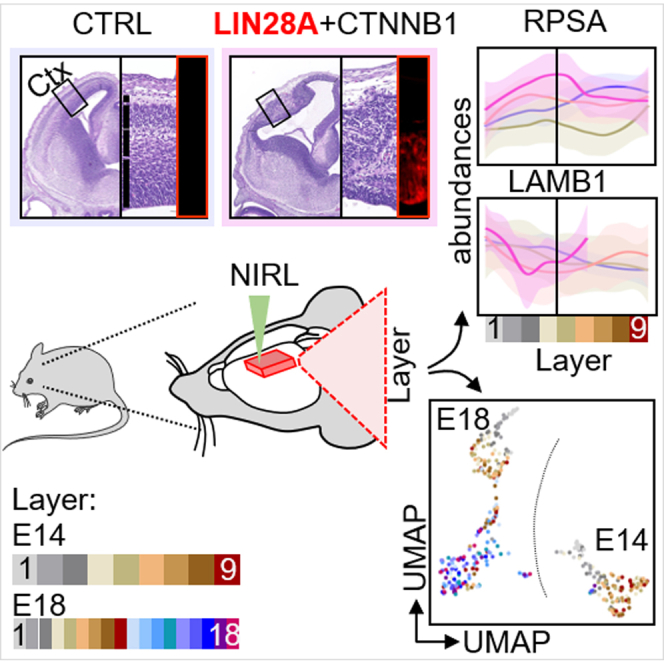

## In Brief

The oncogenic proteins LIN28A and CTNNB1 have been reported in embryonic brain tumors. Their coactivation during brain development did not lead to brain tumor formation, but resulted in disturbed cortical lamination. Spatial proteome patterns of extracellular matrix receptors were altered and accompanied by a porous pial border, neuronal overmigration, and misplaced Cajal–Retzius cells. This phenotype resembles human cobblestone lissencephaly (type 2) and highlights novel implications of the oncogene LIN28A in extracellular matrix integrity.

Cortical brain development is a process that requires precisely controlled spatial and temporal signaling. One such process is the development of the six-layered cerebral cortex (1) including the generation of neural cells (2), differentiation (3), and radial migration (4, 5). Disturbances of these processes can lead to a variety of developmental disorders (6) and to tumorigenesis (7, 8, 9, 10, 11). One pathway, which plays a key role in processes of brain development, is the WNT pathway (12). In canonical WNT-pathway activation, the WNT ligand binds to its receptor Frizzled and the downstream effector protein CTNNB1 enters the nucleus and acts as a transcriptional factor (13). Expression of the stabilized CTNNB1 protein enables its accumulation in the nucleus and transcriptional coactivation of WNT target genes (14, 15). WNT pathway activation is described in early childhood brain tumors, such as in highly aggressive embryonal tumors with multilayered rosettes (ETMRs) arising predominantly in the forebrain. One of the ETMR hallmarks, also used in diagnostics, is the overexpression of LIN28A (16, 17, 18). LIN28A is an RNA-binding protein and a known inhibitor of the let-7 miRNA family, whose components are acting as tumor suppressors (19, 20). By inhibition of the miRNA family, LIN28A maintains proliferation and self-renewal of stem cells during early development (21, 22, 23, 24). LIN28A is expressed in neural tissue from E9.5 and promotes neurogenesis and impedes neuronal differentiation by miRNA regulation (22, 25, 26, 27). The mechanisms leading to an upregulation of LIN28A during tumorigenesis and its specific oncogenic function is not yet fully understood (23). The lacking comprehensive knowledge about ETMR tumor biology impedes the finding of appropriate therapy targets and affected children face a poor prognosis (28). The human glial fibrillary acidic protein (hGFAP)-cre promotor targets GFAP^+^ neural precursor cells in the ventricular zone (VZ) of the cerebral cortex, which have been proposed as cells of origin for ETMR (10, 17, 18, 29, 30). In line with this, ETMR formation has been already successfully shown in hGFAP-positive progenitor cells *in vivo* (29). These neural precursor cells expand first their pool of cells, and then differentiate into radial glial (RG) cells establishing a scaffold for radial migration of neuronal cells to their anticipated locations in the cerebral cortex (4, 5). An *in vivo* model with the sole overexpression of LIN28A in GFAP^+^ neural precursor cells does not lead to tumor formation but results in a transiently higher proliferation rate during embryonic development (31). The sole activation of the WNT pathway in GFAP^+^ neural precursor cells was neither sufficient to drive tumorigenesis but led to a disturbance in cortical lamination (15). The interplay of the WNT pathway with another known developmental pathway, the sonic hedgehog (SHH)–pathway, resulted in the formation of ETMR-like tumors. Based on gene expression data, ETMR are characterized by overexpression of WNT and SHH targets (29, 32). Moreover, let-7-miRNAs were shown to interact with effectors of the SHH pathway (29). Though the exact functional link between LIN28A and WNT and SHH signaling is not known, these data suggest that LIN28A might interact with respective pathways. The characterization of underlying mechanisms is necessary to understand the functioning, to find diagnostic markers and discover novel therapeutic targets. Proteins are the final product and the targetable sites for diverse drugs treatments in cancer and for other diseases (33, 34). RNA-binding proteins (RBP) - like LIN28A - are therefore interesting as they bind mRNA and miRNA thereby affecting the protein turnover at the posttranscriptional level (35, 36). Multiomic analysis of ETMRs with typical LIN28A overexpression display moderate correlation between the transcriptome and proteome (37).

The aim of this study was to investigate if coactivation of LIN28A and the WNT pathway (by expression of a stabilized CTNNB1) in neural precursor cells affect brain development and are sufficient and necessary to initiate tumor formation. We, therefore, generated mouse models expressing each factor alone, or both in combination in hGFAP^+^ precursors and analyzed brain morphology and spatial molecular profiles. As LIN28A and the WNT pathway have important regulatory functions in transcription and translation, our focus was to investigate the protein composition of the cerebral cortex with spatial resolution and to examine structural and functional changes in the forebrain region. Therefore, we used a laser ablation approach with a nanosecond infrared laser (NIRL) system for nano-volume tissue sampling and parallel homogenization with a spatial resolution of about 40 μm per ablated layer (38, 39, 40). We show that the interplay of LIN28A overexpression and stabilized CTNNB1 was not sufficient to initiate tumor growth during embryonal brain development but resulted in spatial disturbances of components of the extracellular matrix (ECM) and morphological changes associated with a lissencephaly type 2–like phenotype.

## Experimental Procedures

### Mouse Models

The animals were kept at 12 h/12 h light/dark cycle with accessible water and food supply. Both male and female mice were examined. All experiments using animals were approved by the local animal care committee (Behörde für Lebensmittelsicherheit und Veterinärwesen in Hamburg N99/2019) and handling was conducted in accordance with local governmental and institutional animal care regulations. The following mouse strains with C57BL/6 background were used: *hGFAP-cre* (30), *|S|-Lin28A(3x)-IRES-eGFP* (29, 31, 41) and *Ctnnb1Δex3* (14, 15). Breeding of the three respective mouse strains resulted in four different mouse models. Littermates containing floxed alleles but no *hGFAP-cre* were referred to as controls (CTRLs). *hGFAP-cre::|S|-Lin28A(3x)-IRES-eGFP* (GL), *hGFAP-cre::Ctnnb1Δex3*^*FL/+*^ (GB), and *hGFAP-cre::|S|-Lin28A(3x)-IRES-eGFP::Ctnnb1Δ ex3*^*FL/+*^ (GBL) models were analyzed during embryonic development at embryonic (E) days E14.5 and E18.5 (Supplemental Fig. S1).

### Brain Volume and Cortical Area Quantification

For the volume quantification, micro-computed tomography scans were performed, with a spatial resolution of 100 μm, at a small animal irradiation platform with SmART+ (Precision X-ray) for freshly dissected brains which were transferred to 1x HBSS (Hank's Balanced Salt Solution) and glucose solution on ice. The X-ray tube for micro-computed tomography (inherent filtration: 0.8 mm Be, additional filtration: 2 mm Al) was operated at a voltage of 40 kV and a current of 8 mA. The 3D modeling and volume quantification was performed with Slicer (version 5.8.1). The volume was measured in three technical replicates and the mean was calculated. Afterward the tissue was fixed in formalin and the brains were dissected to determine the size and weight.

The quantification of the cerebral cortex area was performed with Fiji (ImageJ 2.1.0, https://imagej.net/software/fiji/) (Schneider, Rasband, and Eliceiri 2012), and statistical testing was performed with GraphPad Prism using ANOVA with Tukey’s multiple comparison test (n = 3, *p* < 0.05) (version 9.5.1, GraphPad Software Inc, https://www.graphpad.com).

### Histology, Immunohistochemistry, and Immunofluorescence

Formalin (4%) fixed and paraffin-embedded tissue sections (2 μm) were used for H&E staining and for immunohistochemistry (IHC). The Ventana Benchmark XT machine (Ventana) performed IHC with the following antibodies: BrdU (abcam, ab6326, 1:50), KI67 (abcam, ab15580, 1:100), MAP2C (Sigma-Aldrich, M4403, 1:3000), NeuN (proteintech, 66836-1-Ig, 1:100), pHH3 (Invitrogen Antibodies, MA5-15220, 1:100), Reelin (abcam, ab78540, 1:500), SOX2 (biotechne, AF2018, 0,02 μg/ml), SSTR2 (abcam, ab134152, 1:1000), and TBR2 (abcam, ab23345, 1:200), Caspase-3 (Bio-Techne, AF835, 1:300). Flash-frozen tissue sections (8 μm) were used for IHC with LAMA1 (abcam, ab11575, 1:200). Digitalization was performed with the Hamamatsu Photonics K.K. and NDP.view (version 2.8.24), and further image processing with Photoshop Elements 15 and Fiji (ImageJ 2.1.0, (Schneider, Rasband, and Eliceiri 2012)).

For quantification of SOX2^+^ and TBR2^+^ cells a semiautomated script for Fiji was used (ImageJ 2.1.0, (Schneider, Rasband, and Eliceiri 2012) to determine positive cells within a region interest of the cerebral cortex into 10 bins of 50 μm from the ventricular margin to the superficial cortex layer. The count was performed on technical and biological replicates (n = 4) showing the mean value as heat map performed in in RStudio (version 4.2.3) and as bar plots performed in GraphPad (version 9.5.1, GraphPad Software Inc., La Jolla).

### BrdU Injection and Migration Distance Quantification

Pregnant mice at the embryonic (E) day E14.5 were injected intraperitoneally with 50 mg/kg 5-bromo-2-deoxyuridine (BrdU), and the offspring sacrificed at embryonic day E16.5. IHC of frontal brain sections stained for BrdU and a semiautomated script for Fiji (ImageJ 2.1.0, (Schneider, Rasband, and Eliceiri 2012)) was used for the migration distance quantification of BrdU-positive (BrdU^+^) cells within the cerebral cortex. For the GBL model, we chose a region with clear ventricular border and thinned cortex for comparable analysis. For quantification, the scale was firstly calibrated for the distance measurement based on the image scale. Then the ventricular margin and BrdU^+^ cells were marked manually within the region of interest (ROI). Each distance was quantified automatically from the ventricular margin to the BrdU^+^ cell. To determine the migration distances across the mouse models, we defined 10 bins each covering 50 μm and resulting in a range from 0 to 500 μm. The BrdU^+^ cells were assigned into the respective bin and a two-way ANOVA with Tukey’s multiple comparison test was performed. The cortex thickness was measured three times within each ROI for each replicate. The means of the cortex thickness and migration distances were compared using one-way ANOVA with Tukey's multiple comparisons test. For all statistical test in GraphPad Prism (version 9.5.1, GraphPad Software Inc): n = 3; *p* < 0.05.

To determine the fraction of BrdU^+^ cells re-entering the cell cycle, pregnant mice at E14.5 and E18.5 were injected 1 h before sacrifice with 50 mg/kg BrdU. Using the offspring brains a double-immunofluorescence staining of BrdU and Ki67 was performed (see section “Histology, IHC, and immunofluorescence”). All BrdU+ and BrdU^+^/Ki67^+^ cells were counted within a defined ROI using Fiji (ImageJ 2.1.0, (Schneider, Rasband, and Eliceiri 2012) to calculate the fraction of double-positive cells across the BrdU-postive cell population in biological (n = 2–3) and technical (n = 2–3) replicates. The sum of the replicates was calculated for double-positive (BrdU^+/^Ki67^+^) cells within CTRL, GB, and GBL and the sum of BrdU^+^ cells within the mouse models. Then, the fractions of double-positives were compared between CTRL and GB or CTRL and GBL. Chi-square test was performed with RStudio (version 4.2.3). The results were visualized in GraphPad Prism (version 9.5.1, GraphPad Software Inc).

### Proliferation

IHC of frontal brain sections with pHH3 and Ki67 was used for proliferation quantification. Using Fiji (ImageJ 2.1.0, (Schneider, Rasband, and Eliceiri 2012)), the ventricular margin was marked, and its length was measured. Positively stained cells (cells^+^) were counted along the marked ventricular margin and from the margin 50 μm into the VZ. The margin length and cell count were used to calculate the proliferation rate = cells^+^/100 μm. One-way ANOVA with Tukey’s multiple comparison test (n ≥ 3, *p* < 0.05) was performed in GraphPad Prism (version 9.5.1, GraphPad Software Inc). The same approach of counting positive cells and normalized to 100 μm margin was applied for Cajal–Retzius (CR) cell counting.

### Western Blot

Lysis of fresh frozen tissues with 10% (w/v) homogenates in radio-immunoprecipitation assay buffer (50 mM Tris–HCl pH 8, 150 mM NaCl, 1% NP-40, 0.5% Na-deoxycholate, 0.1% SDS) freshly supplemented with 10x protease inhibitor and PhosStop (Roche, 04693159001) on ice. Total protein content was assessed by Bradford assay (Bio-Rad, #5000205 and #5000206) and a total of 50 μg protein was loaded on the Any kD Mini-PROTEAN TGX Precast Protein Gels (Bio-Rad) or 4 to 12% Mini-PROTEAN TGX Precast Protein Gels (Bio-Rad). After electrophoresis separation, proteins were transferred to nitrocellulose membranes (Bio-Rad, #1620213) by wet blotting. After washing, the membranes were blocked for 1 h with 3% milk (ROTH, T145.2) in PBS for α-dystrogylcan and β-dystroglycan. Five percentmilk in TBS + 1% Tween 20 was used as blocking buffer for the other antibodies. First antibodies: rabbit LIN28A (Cell Signaling Technology, 3978S, 1:250), mouse α-tubulin (Developmental Studies Hybridoma Bank, 12G10, 1:2000), mouse α-dystroglycan (α-DAG) (Merck, 05-298, 1:100), mouse β-dystroglycan (Sant Cruz Biotechnology, sc-165998, 1:400), and mouse Reelin (abcam, ab78540, 1:500). The α-DAG and β-dystroglycan antibodies were diluted with PBS, and the other antibodies were diluted with the respective blocking buffer and incubated overnight at 4 °C. The blots were subsequently washed with PBS. Secondary antibodies: anti-mouse-IgG (Promega, W4028, 1:2500) and anti-rabbit-IgG (Promega, W401B, 1:2500) were diluted as the first antibodies and incubated for 2 h at room temperature. Afterward, washed with distilled water and TBS + 1% Tween 20 and then incubated with the chemiluminescent reagent (Thermo Fisher Scientific, 34577) for visualization. Protein abundance quantification was performed in ImageJ (ImageJ 2.1.0, (Schneider, Rasband, and Eliceiri 2012)). The values were normalized to the respective α-tubulin and one-way ANOVA with Tukey’s multiple comparison test (n = 3, *p* < 0.05) was performed in GraphPad Prism (version 9.5.1, GraphPad Software Inc).

### Samples and Experimental Setting for Spatial Proteomics

Using the NIRL nano-volume sampling system (38, 40), consecutive layers of about 40 μm thickness and an area of 800 μm × 800 μm targeting the forebrain as ROI were ablated and collected from fresh frozen material of embryonic mouse heads from the skin surface to the cortex for mass spectrometry–based characterization with spatial resolution (39). The plume of the ablated layer was collected and transferred in a tube (Protein LoBind Tubes, Eppendorf SE, 0030108116) using 10 μl of 0.01% n-dodecyl β-D-maltoside. For tryptic digestion 20 ng trypsin was added. Technical parameters and all mass spectrometry preparation steps are described in further detail by (39).

### Fraction Library Preparation

One adult brain tissue was conventionally homogenized for building a fraction library to enhance the search results by match between runs function. The tissue was lysed in 1% w/v sodium deoxycholate and 0.1 M triethylammonium bicarbonate buffer (SDC buffer). The sample was boiled at 98 °C for 5 min to denature proteins. Afterward, six cycles with the probe Sonicator at 30% power was applied to destroy DNA and RNA molecules. Protein amount was determined with the bicinchoninic acid test (Pierce bicinchoninic acid Protein Assay Kit, Thermo Fisher Scientific, catalog no. 23227). Then, 100 μg of protein was taken and diluted with SDC buffer to 100 μl. For reduction of disulfide-bonded cysteine residues, 10 mM DTT was added to the sample and incubated for 30 min at 60 °C. Afterward, 20 mM iodacetamid was added and incubated for 30 min at 37 °C in the dark for alkylation of the reduced cysteine residues. Trypsin was added in a ratio of 1:100 trypsin to protein and incubated for 16 h at 37 °C. For trypsin quenching and precipitation of SDC, 100% formic acid (FA) were added to a final concentration of 1% FA v/v. The samples were centrifuged for 5 min at 16,000*g* at room temperature (RT). The supernatant was removed and dried in SpeedVac. Tryptic peptides were resuspended in 100 μl of 10 mM Ambica and an aliquot of 50 μl was taken for basic reversed-phase chromatography (High pH RP-HPLC). The Agilent 1200 series HPLC system (Agilent Technologies, Santa Clara) was used to perform High pH RP-HPLC on a 25 cm ProSwift RP-4H capillary monolithic column (Thermo Fisher Scientific). The peptides were separated in a 45 min run based on their hydrophobicity at a flow rate of 0.2 ml/min with eluent A consisting of 10 mM Ambica and eluent B of 10 mM Ambica in 90% acetonitrile. In total, 13 fractions were attained, then dried in a vacuum centrifuge, and stored at −20 °C until further use. For LC-MS/MS measurement the fractions were previously resuspended in 10 μl of 0.1% FA (39).

### Mass Spectrometry

The LC-MS/MS measurements were performed as previously reported in the study of (39). A Tribrid Mass spectrometer (Orbitrap Fusion, Thermo Fisher Scientific) was coupled to a nano-UPLC (Dionex Ultimate 3000 UPLC system, Thermo Fisher Scientific). Separation and elution of peptides were performed by a linear gradient from 2 to 30% solvent B in 30 min at a flow rate of 0.3 μl/min. Eluting peptides were ionized by using a nano-electrospray ionization source with a spray voltage of 1800 V, and transferred into the mass spectrometry (MS) and analyzed in data-dependent acquisition mode. For each MS1 scan, ions were accumulated for a maximum of 120 ms or until a charge density of 2.0e5 ions (automatic gain control target) was reached. Fourier transformation–based mass analysis of the data was performed with a resolution of 120,000 covering a mass range of 400 to 1300 *m/z*. Precursor selection was set to peptides with charge states between 2+ and 5+ above an intensity threshold of 10,000 for MS2 scans. Fragmentation was performed using HCD (higher-energy collisional dissociation) with a quadrupole isolation window of 1.6 *m/z* and a collision energy of 30%. Data were acquired using an ion trap mass analyser in auto scan range mode with a fixed first mass of 120 *m/z*. Fragment ions were accumulated for 60 ms or to an automatic gain control target of 1.0e4. Already fragmented peptides were omitted for 30 s.

### Data Base Search

The data base search was performed with Proteome Discoverer (version 2.4.1.15, https://www.thermofisher.com/de/de/home/industrial/mass-spectrometry/liquid-chromatography-mass-spectrometry-lc-ms/lc-ms-software/multi-omics-data-analysis/proteome-discoverer-software.html). Spectra were searched using Sequest HT against a reviewed murine SwissProt FASTA database obtained in December 2021 containing 17,085 entries (39). Processing step was performed separately for both measured peptides of laser samples and fraction library with the same workflow. Trypsin was specified as enzyme and two missed cleavages were allowed for the identification in Sequest HT. Further, the carbamidomethylation of cysteine residues was set as a fixed modification. Methionine oxidation and protein N-terminal acetylation were set as variable modification. Peptides with a minimum length of 6 amino acids and a maximum mass of 5000 Da were identified with a precursor mass tolerance of 10 ppm. For matching fragment peaks, the mass tolerance was set to 0.6 Da. For the percolator, the concatenated mode was used with target false discovery rate (FDR) <0.01 for highly confident peptide hits and target FDR <0.05 for peptide hits with medium confidence. Both processed datasets were combined in the consensus step under applying the feature mapper node. For the chromatographic alignment, the maximum RT shift was set to 10 min with a mass tolerance of 10 ppm. For feature linking and mapping, both RT tolerance and mass tolerance were determined automatically. For quantification, unique and razor peptides were considered. Confidence thresholds of the protein FDR validator were set to target FDR <0.01 for highly confident proteins and target FDR <0.05 for proteins with medium confidence. Peptide and protein annotation files are attached (Supplemental Tables S1, S2 and S8).

### Experimental Design and Statistical Rationale

In this study, 4 mouse models were used in biological replicates (n > 3) to analyze the layers of the cerebellar cortex. At E14.5 9 layers were ablated: CTRL (n = 4, 5 hemispheres, 44 samples), GL (n = 3, 3 hemispheres, 27 samples), GB (n = 3, 3 hemispheres, 27 samples), and GBL model (n = 3, 3 hemispheres, 26 samples). For E18.5 mice 18 layers were ablated for CTRL (n = 4, 5 hemispheres, 81 samples), GL (n = 3, 3 hemispheres, 54 samples), GB (n = 3, 3 hemispheres, 54 samples), and GBL (n = 3, 3 hemispheres, 54 samples). The samples D9 (E14.5, GBL) and I10-I18 (E18.5, CTRL) were excluded due to a shift in the ROI during sampling. Sample M4 (E14.5, CTRL) was excluded due to high abundance of human keratins. The additional CTRL replicate was used to ablate the left and right hemispheres as technical replicates and all samples were treated equally (39).

Spatial proteome data analysis was performed in RStudio (version 4.2.3). Of note, mass spectrometry–based data is a technique to investigate relative protein abundances, for simplicity we address it further only as protein abundances.

In total, 367 samples were used for further analysis after the quality check (raw data: Supplemental Tables S3 and S4). The data was log2-transformed and normalized by median subtraction over samples. Each time point (E14.5 and E18.5) was further analyzed separately.

The ablations were conducted on three different days and each batch contained all 4 mouse models and time points, except the additional CTRL samples which were ablated in a separate batch. Batch effect reduction was performed using the BERT framework (version 0.99.16, (42). The mouse models and layers were integrated into classes serving as references. For E14.5: CTRL L1-L4, CTRL L5-L9, GL L1-L4, GL L5-L9, GB L1-L4, GB L5-L9, GBL L1-L4, GBL L5-L9; for E18.5: CTRL L1-L9, CTRL L10-L18, GL L1-L9, GL L10-L18, GB L1-L9, GB L10-L18, GBL L1-L9, GBL L10-L18, and corrected for the batch effect “laser ablation” (LA.batch). Correction for “measurement” (m.batch) was performed without references (data: Supplemental Table S5).

Dimension reduction analysis of the data was performed using Uniform Manifold Approximation and Projection (mixomics package, version 0.2.9.0) with proteins of 100% valid values, and visualized with GraphPad Prism (version 9.5.1, GraphPad Software Inc).

ConsensusClusterPlus (version 1.62.0) was used to define layer clusters based on CTRL samples including proteins with missing values resulting in two clusters for E14.5: C_1_ (layer 1–4) and C_2_ (layer 5–9) and three clusters for E18.5: C_3_ (layer 1–2), C_4_ (layer 3–9) and C_5_ (layer 10–18). Ordering was changed without affecting the cluster results (Fig. 3*D*).

Differentially abundant proteins across layers were analyzed with the limma package (version 3.54.2). For each comparison the replicates of a specific layer (L_n_) were compared to all other layers (L_all-n_) (*i.e.*: L _1_ *versus* L _2 to 9_) using proteins with 70% valid values (*p* < 0.05). For the CTRL model, the top 10 high abundant proteins were selected for each layer. If a protein appeared in the consecutive layer it was excluded for all later layers resulting in the top 10 unique high abundant proteins per layer (first come, first serve principle) (Supplemental Table S6). The unique CTRL layer signatures were then compared across the models layers by relative abundances using a heat map and correlation analysis (corrplot (version 0.92)). The top high abundant proteins were also defined for the GL, GB, and GBL model with the same approach.

Single-sample gene set enrichment was performed using the GSVA package (version 1.42.0, (43)) and applied to the layers of the cortex with the mean proteins abundances of proteins with 100% valid values. For each layer, a z-score was calculated giving the representation of a certain gene set (in total over 500 gene sets) based on the proteins for every layer. Applying linear regression model the gene sets were filtered (*p* < 0.05 and R^2^ > 0.1) and compared across the mouse models.

Gene ontology (GO) analysis for biological processes (BPs) was performed in EnrichR (version 3.2; (44, 45, 46)) for every layer in all models using the significantly high abundant proteins (ordered by log Foldchange, max. 50 proteins). For every search the top 5 terms were selected. All terms were then further condensed using the rrvgo package (version 1.10.0, (47)) to reduce redundant GO:BP terms into parent GO:BP terms. Afterward, for each parent GO:BP term a mouse model and layer deconvolution was performed. The “Best Match” value determines which layer in which model represents the parent GO:BP term the best (highest –log10 of the mean adjusted *p* values for each parent GO:BP term across layer and model (Supplemental Table S7)). The “DiffScore” evaluates the difference of the represented layers across the models for each parent GO:BP term. First, the sum of layers for each model was calculated (*i.e.*: parent GO:BP-term_x_ in CTRL was represented in layer 4, 5, and 7: ∑ = 4+5+7 = 16). If a parent GO:BP term was not present in a model is was assigned with the value −45 (S_9_ = − ([1+9)/2] ∗9)) in E14.5 or −171 (S_18_ = − ([1+18)/2] ∗18)) in E18.5. Then the range was calculated across mouse models (highest sum – smallest sum). A lower “DiffScore” indicates less change of layer representation across models, a higher value indicates a greater change or missing representation.

For each layer cluster (C_1_–C_5_) an analysis of covariance was performed comparing proteins with 70% valid values in CTRL *versus* another mouse model (GL, GB, or GBL). Proteins with significant rate of change (slope) or abundance (intercept) were selected (*p* < 0.05 and R^2^ > 0.1) and categorized into specific proteins for the individual mouse model. These specific proteins were analyzed with EnrichR (version 3.2; (44, 45, 46)) for the top 5 GO:BP terms for BPs (GO:BP). The associated proteins were further categorized as potential interaction partners with LIN28A (line type): direct protein–protein interaction with LIN28A (48) as mRNA targets of LIN28A (35). Additionally, we used a literature miner pipeline (modified version (49)) to evaluate the background knowledge of these proteins associated with the keyword “cortical development.” The proteins were assigned into three categories: 1 = at least one review article; 2 = at least one research article; 3 = no literature found (49).

## Results

### Disturbance of Cortical Lamination but No Tumor Formation

**Fig. 1:**
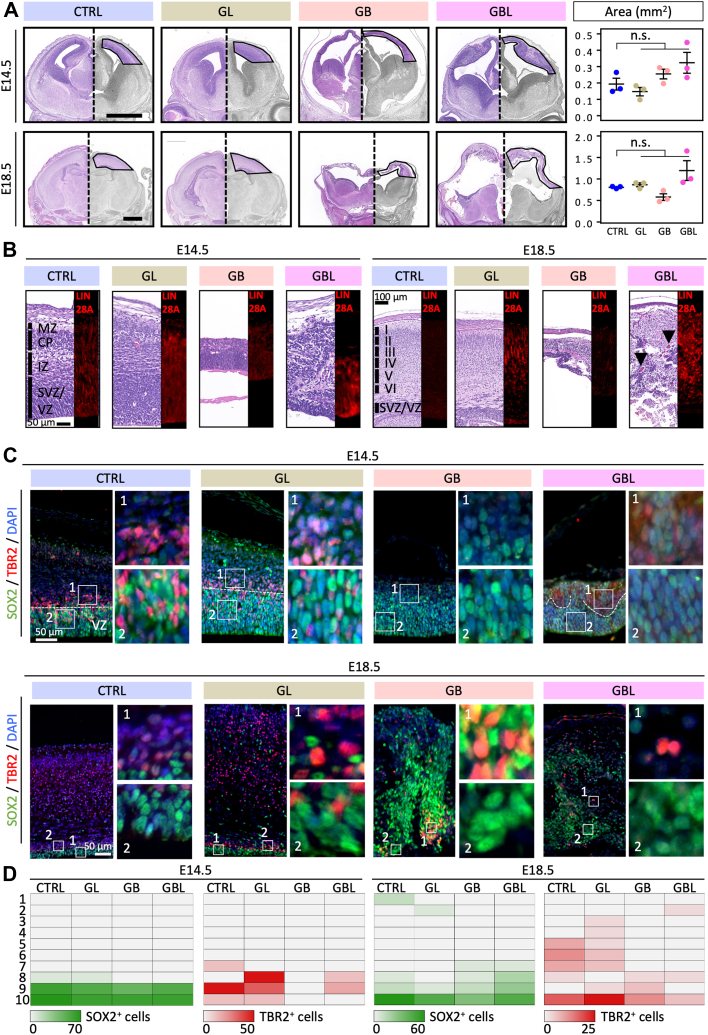
**Coexpression of LIN28A and stabilized CTNNB1 results in severe changes of forebrain histomorphology.***A,* H&E-stained frontal forebrain sections of the embryonic head of control mice (CTRL), transgenic mice overexpressing LIN28A (GL), mice overexpressing stabilized CTNNB1 (GB), and mice coexpressing both factors (GBL) at embryonal days E14.5 and E18.5. In the *right panel*, the cerebral cortex is marked with *dashed lines* for the corresponding area quantification. GB mice display a hydrocephalus and thinned and prolonged cerebral cortex. GBL mice show severely disturbed cortical lamination and a variable cortex thickness. *B,* the magnification of the cerebral cortex (*left*) and the LIN28A immunofluorescence staining (*right*) is shown for each mouse model. *Arrows* indicate larger blood vessels in the brain parenchyma of GBL mice, which are not present in the other models. MZ = marginal zone, CP = cortical plate, IZ = intermediate zone, SVZ = subventricular zone, VZ = ventricular zone, I to VI = cortex layer I to VI. *C,* double-immunofluorescence staining of SOX2 and TBR2 (the scale bar represents 50 μm). The insets show positive cells in the VZ (2) and SVZ (1) at E18.8 and E14.5. *D,* quantification of SOX2^+^ and TBR2^+^ cells. The heat map represents the mean cell count across 10 bins in the cerebral cortex, each bin representing 50 μm.

To analyze the interplay of LIN28A and WNT signaling during embryonal brain development, we generated mice displaying either a sole overexpression of LIN28A (GL), a sole overexpression of stabilized CTNNB1 (GB) or the combination of both (GBL) in hGFAP^+^ neural precursor cells (Fig. 1*A* and Supplemental Fig. S1, *A*–*C*). LIN28A overexpression was validated with LIN28A immunofluorescence staining and respective quantification (Fig. 1*B* and Supplemental Fig. S1, *D* and *E*). While in GL LIN28A was predominately expressed in the cytoplasm of cells of the VZ and CP. In GBL LIN28A expression was additionally found in the nucleus of cells (Fig. 1*B* and Supplemental Fig. S1*E*). In the GB and GBL models, CTNNB1 expression was found in the nucleus indicating activation of the WNT pathway (15) (Supplemental Fig. S1*F*). We evaluated brain histomorphology at embryonic (E) time points E14.5 (shortly after *hGFAP-cre* promotor activation (30)) and E18.5 (shortly before birth, (Fig. 1 and Supplemental Fig. S1*B*). Coactivation of both factors did not lead to brain tumor formation (Fig. 1, *A* and *B*). In line with previous results (31), the GL model did not show coarse morphological changes compared to the CTRL. In contrast, GB and GBL models developed a hydrocephalus and strong disturbances of cortical lamination already apparent at E14.5. The GB and GBL model showed reduced cortical thickness but not reduced cortical area in comparison to CTRL mice (Fig. 1*A*). The brain size, weight and volume of GB and GBL mice were not smaller compared to CTRL (Supplemental Fig. S1*G*). At E18.5 isocortical layering could not be detected in GB and GBL mice. The GBL model additionally displayed variable cortex thickness and large blood vessels in deeper regions around E18.5 (indicated by arrows, Fig. 1*B*) with lethal consequences around the birth time. The cortical marker SOX2 is expressed in the VZ during development (50, 51, 52). In GBL mice, this primary proliferative SOX2^+^ zone was generally established, but highly disturbed compared to the other conditions at E18.5 showing higher scattering in the cerebral cortex (Fig. 1, *C* and *D* and Supplemental Fig. S2, *A*–*C*). In GB mice, we observed a lack of TBR2^+^ intermediate progenitor cells of the subventricular zone (SVZ) at early time points (53). In GBL mice at E14.5 clusters of TBR2^+^ cells were seen in the SVZ-like area. At E18.5 TBR2^+^ cells were also misplaced in superficial layers (Fig. 1, *C* and *D* and Supplemental Fig. S2*A*). Expression of the somatostatin receptor SSTR2 (Supplemental Fig. S2*D*) in migrating cells leaving the VZ (54, 55) was disturbed in the SVZ and intermediate zone with patchy appearance in the GBL model. NEUN, a marker for differentiated neuronal cells (56) showed positive cells in the cortical plate at E18.5 in the CTRL and GL model. The GB and GBL model displayed positive cells but in random distribution with no clear areal specification (Supplemental Fig. S2*B*). The caspase-3 staining revealed apoptotic cells at E18.5 in the GB and GBL model, indicating that cell death may contribute to cortical thinning seen at E18.5 (Supplemental Fig. S2*E*). At E14.5, we did not detect increased cell death in GB and GBL. These results demonstrate that LIN28A overexpression and CTNNB1 stabilization are not sufficient to drive early tumor formation in the brain within this examined time frame but result in disturbed formation of neural cells and disordered lamination.

### Disturbed Cortical Migration and Proliferation

**Fig. 2:**
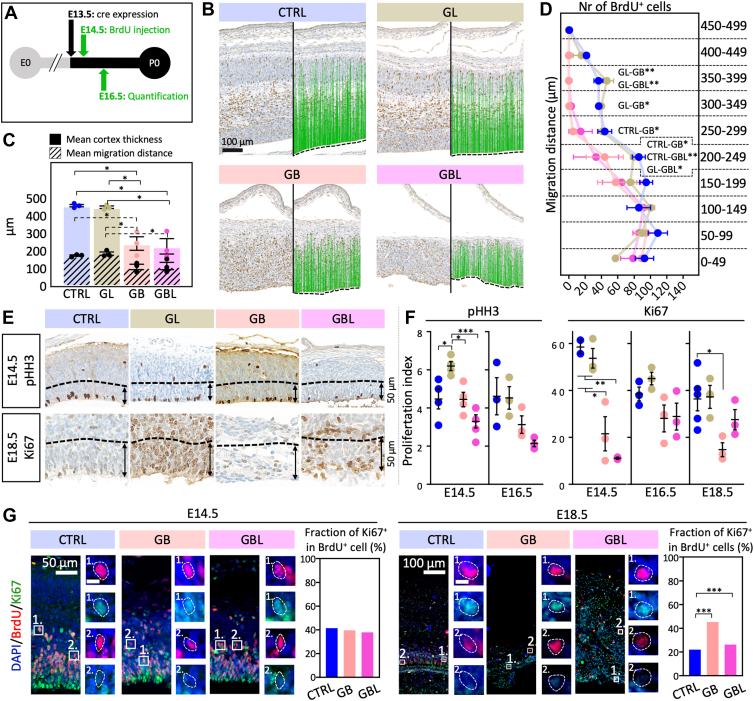
**Alterations of cortical cell migration and proliferation during cerebral development in GL, GB, and GBL mice.***A*, experimental design of bromodeoxyuridine (BrdU) injection and quantification time points. *B,* BrdU staining of frontal sections of cerebral cortices at E16.5 (*left*) and the respective distance quantification output (*right*): *blue lines* indicate the migration distance of BrdU^+^ cells from the ventricular margin (*dashed line*). *C,* mean cortex thickness and mean migration distance across mouse models. Significant differences between models are indicated. *Color filled bars* indicate mean cortex thickness and *dashed bars* indicate mean migration distance (n = 3, one-way ANOVA). *D,* number of BrdU^+^ cells per 50 μm distance bins ranging from 0 to 500 μm across the models. Significant migration differences between models for the respective bins are indicated (n = 3, two-way ANOVA). *E,* immunohistochemical staining of frontal sections of cerebral cortices against proliferation markers pHH3 (E14.5, *upper panels*) and ki67 (E18.5, *lower panels*). *Arrows* indicate the area (spanning 50 μm from the ventricular border) used for quantification of positively stained cells in (*F*). *F,* proliferation index indicates the number of pHH3 or Ki67^+^ cells per 100 μm (n ≥ 3, one-way ANOVA). For *C*, *D*, and *F*: ∗*p* < 0.05, ∗∗*p* < 0.01, ∗∗∗*p* < 0.001, mean and SEM are shown. *G,* BrdU was injected 1 h before sacrifice at E14.5 and E18.5. Costaining of BrdU and Ki67 and quantification of the fraction of double-positive (BrdU^+/^Ki67^+^) cells relatively to BrdU^+^ cells (n = 6, Chi-square test, ∗∗∗*p* < 0.001). Scale bar in the insets indicate 10 μm. pHH3, phosphorylated histone H3.

We hypothesized that disturbed cortical lamination might result from impaired neural migration during cortical brain development. We, therefore, performed cell tracking of BrdU^+^ cells between day E14.5 and E16.5 during embryonic development (Fig. 2*A*). The mean cerebral cortex thickness was significantly thinner in the GB and GBL models within the respective regions of interest (CTRL: $\bar{x}$ = 456.83 μm, GL: $\bar{x}$ = 447.38 μm, GB: $\bar{x}$ = 240.92 μm, GBL: $\bar{x}$ = 225.12 μm, n = 3, *p* < 0.05, Fig. 2*C*) and significantly less BrdU^+^ cells in total were found (CTRL: $\bar{x}$ = 607, GL: $\bar{x}$ = 545, GB: $\bar{x}$ = 380, GBL: $\bar{x}$ = 371, *p* < 0.05). Distance quantification from the ventricular margin (0 μm) to each BrdU^+^ cell in the cerebral cortex (Fig. 2*B*) revealed a significantly reduced mean migration distance in the GB and GBL models compared to the CTRL and GL model (CTRL: $\bar{x}$ = 171.94 μm, GL: $\bar{x}$ = 182.90 μm, GB: $\bar{x}$ = 107.15 μm, GBL: $\bar{x}$ = 112.85 μm, n = 3, *p* < 0.05, Fig. 2*C*). In the GB and GBL models, a significantly smaller number of BrdU^+^ cells were located more distant than 200 μm and no cells were observed to migrate further than 400 μm within the region of interest (Fig. 2*D*).

Previous studies have shown increased transient proliferation in the GL model (31) and a decrease in the GB model (15) during embryonic development. Here, the proliferation rate (cells^+^/100 μm, Fig. 2*F*) was determined based on immunohistochemically stained cortex sections using antibodies against phosphorylated histone H3 (pHH3) and Ki67 for E14.5, E16.5 and E18.5 (Fig. 2*E*). As expected, a transiently higher proliferation rate at E14.5 with pHH3 was observed in the GL model which was abolished at E16.5. In GB, significantly reduced proliferation was detected at E14.5 and E18.5 (Ki67 staining; Fig. 2, *E* and *F*). For the GBL model, a histologically disturbed VZ (Fig. 2*E* and Supplemental Fig. S2) and reduced proliferation was detected in pHH3 (*p > 0.05*) and Ki67 (*p < 0.05*) at E14.5 (Fig. 2*F*). Overall, we detected decreased proliferation rates in the cortex of GB and GBL mice at distinct developmental time points. Though we observed a thinned cortex in the respective genotypes, we did not observe a significant decrease in cortex area (Fig. 1*A*). Therefore, we next asked if LIN28A and stabilized CTNNB1 alter cell cycle dynamics. Therefore, we labelled proliferating cells 1 h before each analyzed time point using BrdU. We then calculated the fraction of cells re-entering the cell cycle (BrdU^+/^Ki67^+^ cells among the total BrdU^+^ cell population). While we have not observed significant differences at E14.5, there was a significantly enhanced fraction of cells re-entering the cell cycle in the GB and GBL model compared to CTRL (Fig. 2*G*; Chi-square test: *p < 0.05*). This indicates that GB mice harbor fewer proliferating cells, but an increased fraction of cells re-enter the cell cycle being in line with previous studies (15, 57). The coactivation of LIN28A re-established proliferation rates as shown with Ki67+ cells at E18.5, together with enhanced cell cycle re-entry. In line with this result, GBL mice showed a trend toward increased cortical area (Fig. 1*A*). In conclusion, stabilized CTNNB1 and the combination with LIN28A resulted in a severe migration disorder and changes in cell cycle dynamics.

### Spatially Resolved Proteomics of the Developing Brain

**Fig. 3:**
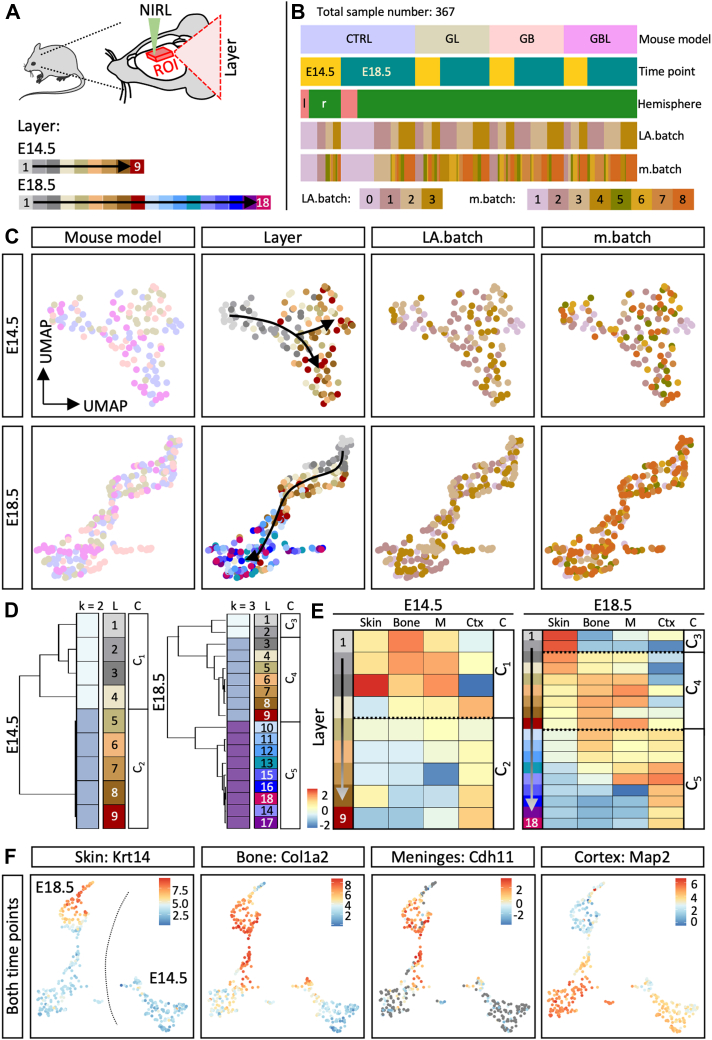
**Spatial proteome analysis of the murine cortex of CTRL, GL, GB, and GBL mice.***A,* experimental design for spatial proteomics using the nanosecond infrared laser (NIRL) ablation system to ablate consecutive layers (∼40 μm) directly from the frozen sample targeting the region of interest (ROI) within the forebrain region as previously described (39). At E14.5, nine consecutive layers were ablated and at E18.5 18 layers were ablated. *B,* in total, 367 samples were obtained, including CTRL (n = 4), GL (n = 3), GB (n = 3), and GBL (n = 3) for each time point E14.5 (124 samples) and E18.5 (243 samples). The samples were ablated and further processed in four batches (LA.batch) and mass spectrometric measurements were performed in eight batches (m.batch). *C,* uniform manifold approximation and projection (UMAP) for the E14.5 and E18.5 dataset based on proteins with 100% valid values. Legends in (*A* and *B*) apply for color scheme in (*C*). *D,* consensus clustering analysis combines individual layers into layer clusters: C_1_ and C_2_ for E14.5 and C_3_–C_5_ for E18.5. k = number of clusters, L = layer, C = cluster. *E*, Mean abundance (column scaled) of marker proteins for skin (FLG, KRT14, LORICRIN; (58)), bone (COL1A1, COL1A2, SERPINF1; (59)), meninges (=M; CDH11, CRABP2, TAGLN; (58, 60)), and cerebral cortex (=Ctx; TBR1, MAP2, BCL11B; (58)). C = cluster C_1_–C_5_ are indicated with *dashed lines*. *F,* abundance of marker proteins for skin (KRT14), bone (COL1A2), meninges (CDH11), and cortex (MAP2) mapped onto the UMAP with samples from both time points using proteins with 100% valid values. CTRL, control.

In order to elucidate the molecular mechanisms resulting in the observed phenotypes, we aimed to analyze molecular parameters in a spatially resolved manner. As LIN28A and CTNNB1 are both transcriptional and translational regulators, we aimed to investigate the resulting protein level using mass spectrometry–based proteome data with high spatial resolution. Therefore, we used the NIRL nano-volume sampling system (38, 40) and ablated consecutive cortical layers directly from the skin surface into the cerebral cortex, as described previously (39). We ablated nine consecutive layers at E14.5 and 18 consecutive layers at E18.5 of the CTRL condition in biological replicates (n = 4) and the mouse models GL, GB, and GBL (n = 3) (Fig. 3*A* and Supplemental Fig. S3*A*). The layers were ablated in four batches (LA.batch) resulting in a data set with in total 367 samples measured in eight batches (m.batch) and identifying over 5000 proteins (Fig. 3*B*). The initial sample distribution was mainly driven by developmental differences according to the time points (Supplemental Fig. S3*B*). Hence, downstream analysis was done separately for E14.5 (124 samples) and E18.5 (243 samples) (Supplemental Fig. S3*C*). By applying the batch effect reduction algorithm BERT ((42), we reduced technical batch effects and preserved over 3000 proteins providing quantitative information for comprehensive analysis. Dimension reduction analysis based on 100% valid values showed a gradient distribution from superficial to deeper layers by the represented proteins (Fig. 3*C*). To better understand similarity across layers, we performed consensus clustering based on the CTRL samples and detected two clear layer clusters at E14.5 (C_1_ and C_2_) and three clusters at E18.5 (C_3_–C_5_) (Fig. 3*D* and Supplemental Fig. S4*A*). Marker proteins for the skin (58), bone (59), meningeal structures (58, 60), and cerebral cortex (58) allowed to determine anatomical identities of the ablated layers (Fig. 3*E*). Layers belonging to the clusters C_1_, C_3_, and C_4_ showed higher abundances of proteins associated with the skin, bone, and meningeal structures (Fig. 3*E*). The clusters C_2_ and C_5_ showed higher abundances of cerebral cortex markers (Fig. 3*E*) and were differently expressed across the GL, GB, and GBL layers (Supplemental Fig. S4*B*). Additionally, we have confirmed that the abundance of the marker proteins was higher at E18.5 than in E14.5 corresponding to the structural maturation (Fig. 3*F* and Supplemental Fig. S5, *A* and *B*). Taken together, spatially resolved proteomics distinguished cortical layers and revealed disturbances in the GL, GB, and GBL models, as compared to the CTRL.

### Characterization of Unique Layer Signatures

**Fig. 4:**
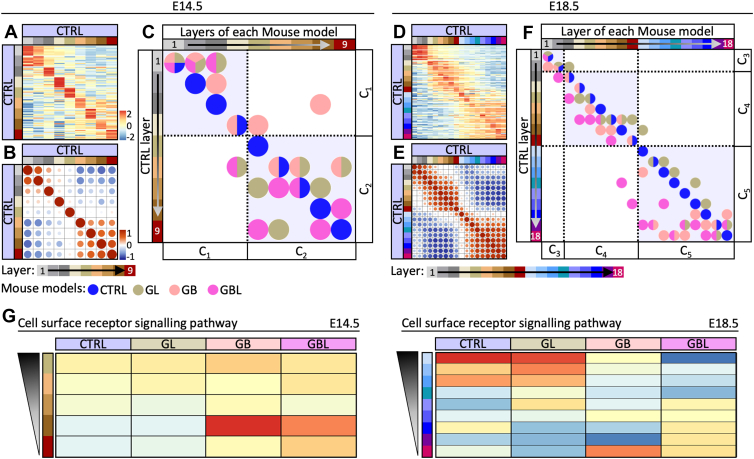
**Spatial representation of CTRL layer signature proteins across mouse models.***A* and *D,* heat map representing the top 10 high abundant proteins (Supplemental Table S6) of each CTRL layer (*y*-axis) in all CTRL layers (*x*-axis) at E14.5 (*A*) or E18.5 (*D*). Scaled rows. *B* and *E,* correlation analysis based on the top 10 high abundant proteins of each CTRL layer at E14.5 (*B*) or E18.5 (*E*). *C* and *F,* summary of correlation analysis results based on the top 10 high abundant proteins of each CTRL layer. *Dots* represent the highest positive correlation for each CTRL, GL, GB, and GBL layer (*x*-axis) compared to the CTRL layer (*y*-axis). Layer clusters C_1_–C_5_ are indicated with *dashed lines*. At E18.5, superficial GBL layers show a shift of protein patterns toward deeper layers of the CTRL. *G,* gene set “cell surface receptor signaling pathway” showing linear decrease over layers in both time points (E14.5 and E18.5) of the CTRL cortex. Representation across the mouse models is shown as heat map. CTRL, control.

We further characterized a unique signature for each CTRL layer based on the unique top 10 high abundant proteins for each layer (Supplemental Table S6). Correlation analysis among the CTRL samples demonstrates that these unique top 10 high abundant proteins are sufficient for layer characterization (Fig. 4, *A*, *B*, *D*, and *E* and Supplemental Fig. S6*A*). The top 10 high abundant proteins were detected in all mouse models, but the characteristic pattern observed in the CTRL condition was abolished among the GL, GB, and GBL layers (Supplemental Fig. S6, *B*–*E*). To determine the highest similarity of cortical layers in the GL, GB, and GBL models compared to the CTRL, we selected the highest correlation value of each layer comparison (Supplemental Fig. S6, *C* and *E*). Compared to CTRL, most layers of GL mice fell into similar layers or the same layer cluster (C_1_–C_5_, Supplemental Fig. S6, *B* and *E*). In contrast, deeper layers in the GB model (E14.5, *x*-axis: C_2_) were more similar to superficial CTRL layers (*y*-axis: C_1_). In the GBL model (E18.5) the superficial layers (*x*-axis: C_1_ and C_2_) resembled deeper CTRL layers belonging to C_4_ and C_5_ (*y*-axis) (Fig. 4, *C* and *F*). In line with this, SOX2^+^ and TBR2^+^ cells were scattered in more superficial cerebral cortex areas in the GBL model (Fig. 1, *D* and *E* and Supplemental Fig. S2*A*). Additionally, we have performed single-sample gene set enrichment analysis to find gene sets which fit linear representation along the cortex-related layers at both developmental time points (E14.5: layer 5–9; E18.5: layer 10–18). We have compared trend lines which are in the same direction (positive correlation) or opposite (negative correlation) direction in E14.5 and E18.5 and investigated the changes across the mouse models. Distinct spatial gene set enrichment gradients changed inversely at different time points of development (*e.g.,* homeostatic processes), while others were similarly distributed over time (*e.g.,* macromolecule localization) (Supplemental Fig. S7). Moreover, functional gene set enrichment gradients were changed in GB and GBL models. A drastic change for the cell surface receptor signaling pathway was seen in the GBL model (Fig. 4*G*). This is well in line with the severe phenotype of layer disruption.

Generally, all mouse models had a characteristic unique layer signature capturing the differences across layers, while the overlap of the top 10 high abundant proteins among the mouse models were greater in the superficial layers (Supplemental Fig. S8). GO analysis for biological processes (condensed into parent GO:BP terms to reduce redundant terms, Supplemental Table S7) indicated functions that are represented similarly across the models (E18.5: “skin development”). Other functions appear rather in the recombinant models GL, GB, and GBL (E14.5: “gene expression”). Some terms show a greater shift of the representing layers from superficial layers in the CTRL to the deeper layers in the GBL model (E14.5: “epidermis development;” E18.5: “cellular response to monoamine stimulus,” Supplemental Fig. S9). In summary, the GB and GBL models showed differences in spatial protein abundances. The GBL displayed a shift in layer identity with superficial layers being more similar to deeper layers of the CTRL.

### Imbalanced Protein Abundances Associated with a Lissencephaly-Like Phenotype

**Fig. 5:**
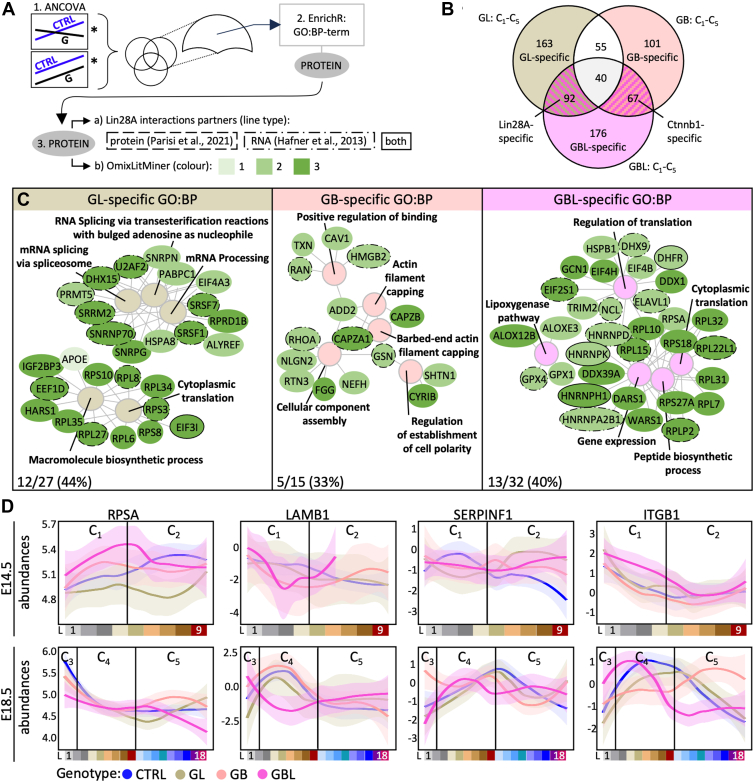
**Layer and mouse model–specific protein representation reveals ribosomal proteins and extracellular matrix components to be differentially distributed in brains with combined CTNNB1 stabilization and LIN28A overexpression coactivation.***A,* workflow analyzing proteins of interest considering spatial resolution and mouse models. Proteins with significant difference upon ANCOVA based regression analyses (∗) were taken as specific proteins for each mouse model after generating a Venn diagram. These proteins were further analysed using EnrichR (44, 45, 46)) for gene ontology terms for biological processes (GO:BP term). The associated proteins were then categorized by (a) known LIN28A interaction patterns (indicated by line type) and (b) the background knowledge using a literature mining tool (modified version (49)) using the keyword “cortical development” and its respective categories: 1 = at least one review paper, 2 = at least one research paper, 3 = no further literature available. *B,* Venn diagram of significant proteins after ANCOVA between CTRL *versus* GL or GB or GBL for all layer clusters (C_1_–C_5_). *C,* top 5 GO:BP terms for each model-specific protein collection. Proteins are colored based on the OmixLitMiner categories. The line types indicate previously described interaction with LIN28A. The *percentage* shows the proportion of potential partners to the total number of proteins for each panel (*bottom corner*). *D,* spatially resolved protein abundances of extracellular matrix components and their respective receptors. L = layer, C_1_–C_5_ = defined layer clusters based on consensus clustering analysis in CTRLs. ANCOVA, analysis of covariance; BP, biological process; CTRL, control; GO, Gene ontology.

In order to capture specific changes in protein abundance in a spatial context, we performed analysis of covariance for each layer cluster separately (C_1_–C_5_) and compared the CTRL conditions with the other mouse models (GL, GB, or GBL, Fig. 5*A*). Specific proteins for GL, GB, or GBL with a significantly different rate of change (slope) or abundance (intercept) compared to the CTRL (*p* < 0.05 and R^2^ > 0.1, Fig. 5, *A* and *B*) in either cluster were further described using GO analysis for biological processes (GO:BP terms, Fig. 5*C*). GL-specific terms were predominantly related to RNA processing similarly to the LIN28A-specific terms (Supplemental Fig. S10) while in the GBL model, the terms were related to translation processes, and were associated with different proteins. The GB model was defined by distinct GO:BP terms, such as cell polarity and cellular component assembly and shared similarities to the CTNNB1-specific terms (Fig. 5*C* and Supplemental Fig. S10). Of note, the LIN28A-, GL- and GBL-specific terms had a high proportion of altered proteins that were reported as direct interaction partners of LIN28A at protein or mRNA level, potentially affecting their translation (>40%, Fig. 5*C*) (35, 48). We further categorized the proteins using a literature mining tool (modified version (49)) to highlight potential proteins of interest (green color code in Fig. 5*C* distinguishing between categories: 1 = at least one review paper/well known, 2 = at least one research paper/known, 3 = no further literature available/not known in context of “cortical development” as keyword (49)). Beyond others, the laminin receptor protein RPSA was significantly altered in the GBL model. RPSA was shown to be involved in radial glia morphology shaping and affects cortical migration (61). We therefore analyzed the spatial resolved abundances of RPSA in more detail and further focused on known associated factors like laminin (LAMB1), the ligand Serpinf1 (PEDF) (61) and integrins (ITGB1, Fig. 5*D*). The factors showed a strong deviation in the GBL model. We particularly observed a striking drop of LAMB1 in the C_1_ and C_4_ layers, representing meningeal structures, within the GBL model, but not in the other models (Fig. 5*D*).

**Fig. 6:**
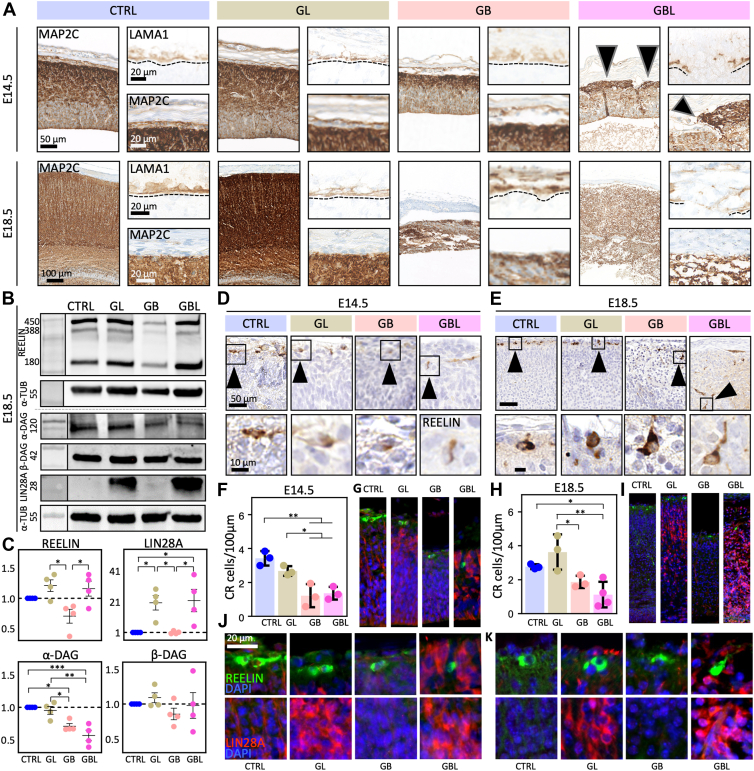
**Porous pial border and neural overmigration in GBL mice.***A,* immunohistochemistry against laminin (LAMA1) as pial marker and against MAP2C as dendritic marker for neuronal tissue. Pial border is indicated by *dashed line*. Ectopic neuronal tissue located above the pial border in the GBL model is indicated by *arrows*. *B,* Western blot of cortical lysates stained against Reelin (450, 388, 120 kDa), α-DAG (α-dystroglycan, 120 kDa), β-DAG (β-dystroglycan, 42 kDa) and LIN28A (28 kDa) at E18.5. α-TUB (α-tubulin, 55 kDa) was used as housekeeping protein for normalization. *C,* respective quantification of Western blot signals (*B*) based on normalized values (n ≥ 3, one-way ANOVA, ∗*p* < 0.05, ∗∗*p* < 0.01, ∗∗∗*p* < 0.001; mean and SEM are shown). Reelin levels were decreased in the GB model compared to GL and GBL. *D* and *E,* immunohistochemical staining against Reelin in E14.5 and E18.5. Magnified Cajal–Retzius (CR) cells are indicated by *arrows*. In GBL mice, Cajal–Retzius (CR) cells were located in deeper brain regions. *F* and *H,* quantification of CR cells/100 μm (n ≥ 3, one-way ANOVA, ∗*p* < 0.05, ∗∗*p* < 0.01, and ∗∗∗*p* < 0.001; mean and SEM are shown). *G* and *I,* double-immunofluorescence of LIN28A and Reelin showing insets of positive cells at E14.5 (*J*) and E18.5 (*K*).

Immunohistochemical investigation of LAMB1/LAMA1 expression revealed a porous pial border in the GBL model at (E14.5) with aggregations of neuronal tissue (MAP2 positive) (62) above that border (Fig. 6*A*). At E18.5, no clear pial border could be observed in GBL with larger blood vessels in deeper cortical regions (Figs. 1 and 6*A*). The overmigration phenotype observed in the GBL model resembles the phenotype of the human migration disorder lissencephaly type 2, which is associated with hypo-glycosylation of α-DAG (63). We therefore analyzed α-DAG levels and detected a significant reduction in the CTNNB1-associated GB and GBL models at E18.5, but no significant difference in the β-dystroglycan (Fig. 6, *B* and *C* and Supplemental Fig. S11, *A* and *B*). The impaired and disturbed cortical migration phenotype of the GB model is similar to the phenotype of the human disease lissencephaly type 1, which is characterized by reduction of Reelin, an important regulator of radial migration, mainly expressed by CR cells (64, 65, 66, 67). At E14.5 Reelin expression was significantly reduced in the GB model compared to the GL and GBL model with elevated Reelin abundance (*p* < 0.05, Supplemental Fig. S11, *A* and *B*). Histological observation showed ectopically located CR cells in deeper layers of the cortex in the GBL model (Fig. 6, *D*, *E*, *G*, *I*–*K*). The number of CR cells was reduced in the GB and GBL models, suggesting that the number of CR cells and Reelin expression do not correlate positively in the GBL model (Fig. 6, *B*, *C*, *F*, and *H*). Double staining revealed that LIN28A and Reelin were not colocalized in the same cells. This underlines that Reelin^+^ cells are not targeted by the hGFAP promotor and indicates an indirect effect of LIN28A onto Reelin expression in GL and GBL models (Fig. 6, *G*, *I*, *J*, and *K*). In summary, expression of stabilized CTNNB1 resulted in a lissencephaly type 1–like phenotype with reduction of Reelin levels, whereas a coactivation of both LIN28A overexpression and stabilized CTNNB1 rescued the Reelin levels but led to a porous pial border and mislocalized CRs resulting in a lissencephaly type 2–like phenotype.

## Discussion

In this study, we investigated the interplay of the developmental factor LIN28A and the WNT pathway key player CTNNB1, which are both described as oncogenic features in ETMRs (16, 17, 18, 29). An established mouse model for ETMR is initiated by coactivation of the WNT and SHH pathways but lacks the hallmark feature of LIN28A overexpression (29). Therefore, we aimed to develop an ETMR mouse model displaying LIN28A overexpression. We show here that the coactivation of LIN28A overexpression and stabilized CTNNB1 in neural precursor cells during embryonic development, defined as the GBL model, was not sufficient to drive tumor formation during the examined time frame of embryonal development. Instead, we observed histomorphological and molecular features resembling the human disorder cobblestone lissencephaly type II (68) with porous pial border and neuronal overmigration starting from E14.5. Using spatial proteomics, we show a shift in cortical layer identities with a change of the ECM composition revealing new functions of LIN28A during brain development.

The RNA-binding protein LIN28A is an oncogenic factor being overexpressed in various cancer types occurring in the brain, ovary, breast, intestine, or colon (17, 18, 69, 70, 71). The embryonal brain tumor ETMR expressing LIN28A also displays additional features, such as WNT and SHH pathway activation (17, 18, 29). However, a sole LIN28A overexpression in neural precursor cells does not lead to brain tumor formation (31), suggesting that other factors are required to initiate tumor growth. CTNNB1 is an effector of the WNT signaling pathway which has been reported to be mutated in various cancers, including ETMR (17, 18, 29, 72, 73). The initial intention of combining LIN28A with stabilized CTNNB1 was to create a preclinical model for ETMR, a tumor that usually occurs in very young infants (0–3 years in humans). A previously developed ETMR model based on the hGFAP-cre promotor correspondingly displayed tumor formation already at E18.5 (10, 29). Using the same promotor, we did not see any tumor formation at this time point in GBL mice. As a limitation, GBL mice could not be analyzed at postnatal stages and a potential brain tumor formation cannot be ruled out at later time points. In other cancer types, such as intestinal cancer, LIN28A and its paralog LIN28B drive tumor formation, and cooperate with activated *Ctnnb1* (74), suggesting cell context–dependent oncogenic functions.

Previous studies investigating LIN28A overexpression demonstrated increased transient proliferation during early embryonic development (31, 75). Similarly, CTNNB1 has been described to increase the neural precursor pool during brain development by enhancing cell cycle re-entering and resulting in a thinned, but horizontally enlarged cortical surface area and ventricle enlargement (15, 57). In line with this, we observed enhanced cell cycle re-entry in GB and GBL mice at E18.5 and increased total brain volume and comparable cortical area (Fig. 1*A* and Supplemental Fig. S1*G*). The increased head and brain size underlines that the GB and GBL models do not display a microcephalic phenotype. GBL mice displayed a thicker cortex than GB mice but a histologically disturbed VZ and cortical lamination. This might indicate an interaction between both factors. Of note, *LIN28A* was described to be regulated by the WNT pathway as a direct target of CTNNB, which results in increased LIN28A mRNA levels in breast cancer, adult mammalian retina, and mouse hippocampus and promotes proliferation (76, 77, 78). However, we did not detect a significant increase of LIN28A protein levels in GB mice (Fig. 6*C*). Additionally, CTNNB1 itself might be regulated at the mRNA level by LIN28A (35) and reform its downstream function. Proliferative cells need sufficient supply ensured by biogenesis of required components. LIN28A promotes genes involved in ribosome biogenesis (48) and dysregulation of ribosomal proteins is described in different cancer types supporting the proliferative behavior (79, 80). We showed that both LIN28A overexpressing GL and GBL models show dysregulated proteins belonging to the ribosomal family (Fig. 5), which have been described as potential LIN28A interaction partners (35, 48). Nevertheless, the proteins associated with these terms were different in the GL and GBL model indicating context-dependent functional dynamics.

In addition to changes in progenitor cell proliferation, CTNNB1 stabilization was strongly related to migration disturbances and disordered lamination in the GB and GBL models underlining that the WNT pathway affects migratory neurons (81, 82). The disturbed migration was also represented in the proteomic signature. The GB model showed protein abundance changes in components involved in actin capping and filament assembly related to impaired cell motility (Fig. 5 and Supplemental Fig. S10) (83).

Although, the mouse brain is intrinsically lissencephalic lacking brain convolutions, mice have been proven to be a useful model to study neuronal migration disorders outweighing this limitation (65, 66, 84). The human neuron migration disorder lissencephaly type I is mainly driven by mutations of LIS1, a condition closely mimicked in the *reeler* mouse model (65, 66, 85, 86, 87, 88). Similar to the well-established *reeler* phenotype induced by knockout of the *Reelin* gene in mice (84), the GB model showed abnormal cortical lamination featured by reduced REELIN expression and number of CR cells resembling also implicating similarities to human lissencephaly type I (Fig. 6, *B*–*E*). The interaction between the Reelin signaling pathway with downstream components like DAB1 and LIS1 affects cortical development and neuronal migration (89). LIS1 itself plays a major role in neuronal migration and proliferation during brain development (85, 90, 91). It regulates dynein motor-protein localization and modulation of microtubules (90, 92). Microtubules are also relevant for ECM-dependent neuronal migration and spindle orientation during symmetric division of neuroepithelial cells (90, 91). Patients harboring a *Reelin* mutation causing reduced Reelin protein abundance develop the migration disorder lissencephaly with cerebellar hypoplasia (93). Generally, during development of the cerebral cortex, Reelin is mainly secreted by CR cells in the marginal zone regulating radial migration of neurons along the RG cells and fostering proper cortical lamination (64, 65, 66). In contrast to the GB model, coactivation of CTNNB1 stabilization and LIN28A overexpression in GBL mice resulted in a severe migration disorder but increased Reelin expression was observed. Additionally, its receptor ITGB1 (94, 95, 96, 97) was spatially dysregulated in the GBL model at E18.5. Reelin-expressing CR cells responsible for proper radial migration were mislocalized in deeper cortical regions in the GBL model. The CR cells themselves are led along the meninges by secreted factors to the marginal zone (98). At E14.5, we observed CR cells localized at the cortical surface indicating that CR cells reached their anticipated location, but disruption of the pial border and neural overmigration displace CR cells to deeper cortical regions. The neuronal migration disorder is known as the human disease cobblestone (type II) lissencephaly (68). Mutation in glycosyltransferases like FKRP and POMT1 (99, 100) lead to hypoglycosylation of the α-DAG and thereby fail to interact with the ECM resulting in a disturbed pial border (63). These glycosyltransferases were not detected in the mass-spectrometry-based proteome data, but we show that both CTNNB1 related models, GB and GBL have reduced α-DAG levels (Fig. 6, *B* and *C*), indicating that this factor alone is not sufficient to disturb the pial border leading to neuronal overmigration. While certain features in the GBL model resemble the human lissencephaly disorder, the lissencephaly spectrum involves a wider range of mechanistic disruption. Laminin deficiency was also shown to cause congenital muscular dystrophy and often developing cobblestone lissencephaly and eye malformations (101, 102). These effects and the effects of glycosyltransferases in the GBL model have to be further investigated.

Since LIN28A—as a RNA-binding protein—has the potential to alter the resulting proteome composition as shown in LIN28A expressing ETMRs, where the proteome signatures significantly differ from their transcriptome profiles (37) we aimed to find proteins which explain the observed phenotype. We ablated layers from the skin surface into the cerebral cortex during embryonic development (E14.5 and E18.5) and provide this data for comprehensive analysis of the spatially resolved proteome in the CTRL and other mouse models. The ablated layers revealed individual protein signatures in the cerebral cortices and clear spatial patterns (Fig. 4). Future refinement of this method will allow to gain further insight with increased spatial resolution. The layer signatures in the GBL model, revealed a lack of “positive regulation of cell-substrate adhesion” at E14.5 (Supplemental Fig. S8). Later at E18.5, a shift in layer identity was observed. Superficial layers in the GBL model resembled deeper CTRL layers associated with the cerebral cortex indicating overmigration based on the proteomic signature. The “cell surface receptor signaling pathway” was increased in superficial layers in the CTRL but was represented inversed in the GBL model indicating a disturbed interaction with the pial basement membrane. The GBL showed distinct proteins altered compared to GL- and LIN28A-specific terms, indicating a novel function in the context of coactivation with CTNNB1 (Fig. 5*C* and Supplemental Fig. S6). High spatial deviation of the proteins related to the ECM and their receptors was observed in the GBL model compared to the other models. The ECM protein LAMININ (LAMB1/LAMA1) (103) was decreased corresponding to the disturbed pial border. The LAMININ receptor RPSA (104) appeared as another interesting candidate which was dysregulated in the GBL model. It was shown that a knockdown of *Rpsa* leads to malformation of RG cells affecting the radial migration process (61). This led to the hypothesis of improper integration of RG and CR cells due to accumulation of imbalances at the receptor and ligand level. In future studies it should be investigated how the altered molecular features fail the maintenance of the pial border and result in a severe form of overmigration in the GBL model (Supplemental Fig. S12). We have observed that LIN28A is mainly located in the cytoplasm in the GL model while it is also found in the nucleus in the GBL model (Supplemental Fig. S1*E*). A possible synergistic interaction between LIN28A and CTNNB1 has to be further analyzed. One approach would be single-cell or single-nucleus transcriptomic analysis. This would allow an insight on cell population composition across the models and cellular differences affecting differentiation and cortical development. We observed a lack of TBR2^+^ cells in the GB and positive clusters of TBR2^+^ cells in GBL (Fig. 1*B*). This might explain the various thickness in the GBL model as this progenitor population is responsible for cortical expansion (105). This could be an interesting aspect of further investigation using, for example, lineage tracing. Another aspect is the role of RNA-binding proteins and the mechanism of posttranscriptional regulation. Similar to the RNA-binding protein LIN28A, LIS1 has RNA-binding functions (106). Overexpression of LIS1, which is associated with lissencephaly type I disorder when mutated, was shown to increase ECM components (106).

In summary, we show that in the GBL model LIN28A counterparts some effects of CTNNB1 preserving Reelin expression and stabilizing the proliferation rate despite histological disturbances in the VZ and cortical layering. Moreover, LIN28A affects translation of ribosomal components and components of the ECM revealing potential novel roles. Finally, the GBL model shows similarities with human lissencephaly type 2 and may be suitable for further analyses revealing the molecular foundations of this severe migration disorder.

## Data Availability

Full LC–MS/MS data are available *via* ProteomeXchange with identifier PXD053649.

## Supplemental data

This article contains supplemental data.

## Conflict of interest

The authors declare no competing interests.
