# Supplementary material for: Spatial Proteomics Reveals Distinct Protein Patterns in Cortical Migration Disorders Caused by LIN28A Overexpression and WNT Activation
Source: Mol Cell Proteomics. 2025 Jul 16;24(9):101037. doi: 10.1016/j.mcpro.2025.101037 (PMC12419088; doi:10.1016/j.mcpro.2025.101037)
Supplement: Supplementary Figures [file mmc9.pdf]

Navolić et al., Fig. S1

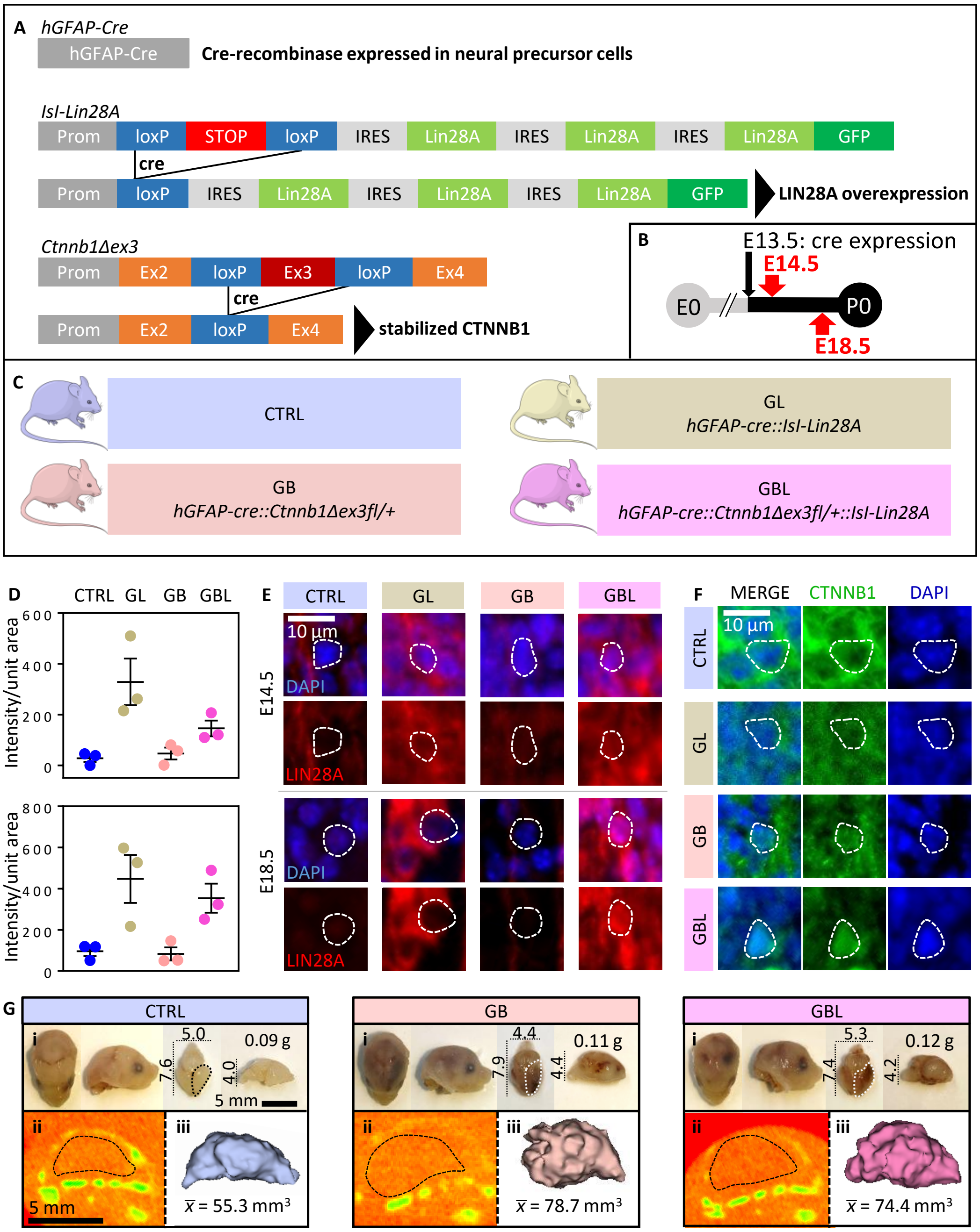

Fig. S1: Scheme of transgenes and resulting mouse models.
(A) Cre-Recombinase expressed in *hGFAP*-positive neural precursor cells leads to removal of a functional STOP sequence in the *Isl-Lin28A* construct. This results in LIN28A overexpression. Cre recombination of the *Ctnnb1Δex3* construct removes exon3 resulting in stabilized CTNNB1. **(B)** *hGFAP*-dependent Cre recombination initiates at embryonic (E) day E13.5 (Zhuo et al. 2001). The time points E14.5 and E18.5 were investigated during development. **(C)** Breeding of the three respective transgenic mouse strains resulted in four different mouse models: CTRL = control condition containing one or both floxed constructs but no Cre (= no recombination event), GL = overexpression of LIN28A in hGFAP^{+} cells, GB = stabilized CTNNB1 in hGFAP^{+} cells, and GBL = overexpression of LIN28A and stabilized CTNNB1 in hGFAP^{+} cells. **(D)** Quantification of Lin28A intensity per unit area. **(E)** Immunofluorescence staining showing localization of LIN28A in the cytoplasm and nucleus. Scale bar represents 10 µm in all panels. **(F)** Immunofluorescence staining of CTNNB1 showing the nuclear aggregation in the GB and GBL model. Scale bar represents 10 µm in all panels. **(G)** Images of heads of E18.5 mice and the dissected brains for CTRL, GB and GBL. Size measurements in (i) for all genotypes are presented in mm. (ii) Sagittal plane of micro-CT scans, the dashed line indicates the brain region. (iii) 3D volume reconstruction of the brain with mean brain volumes (n=3). The scale bar in (ii) represent 5 mm for panels (ii) and (iii).

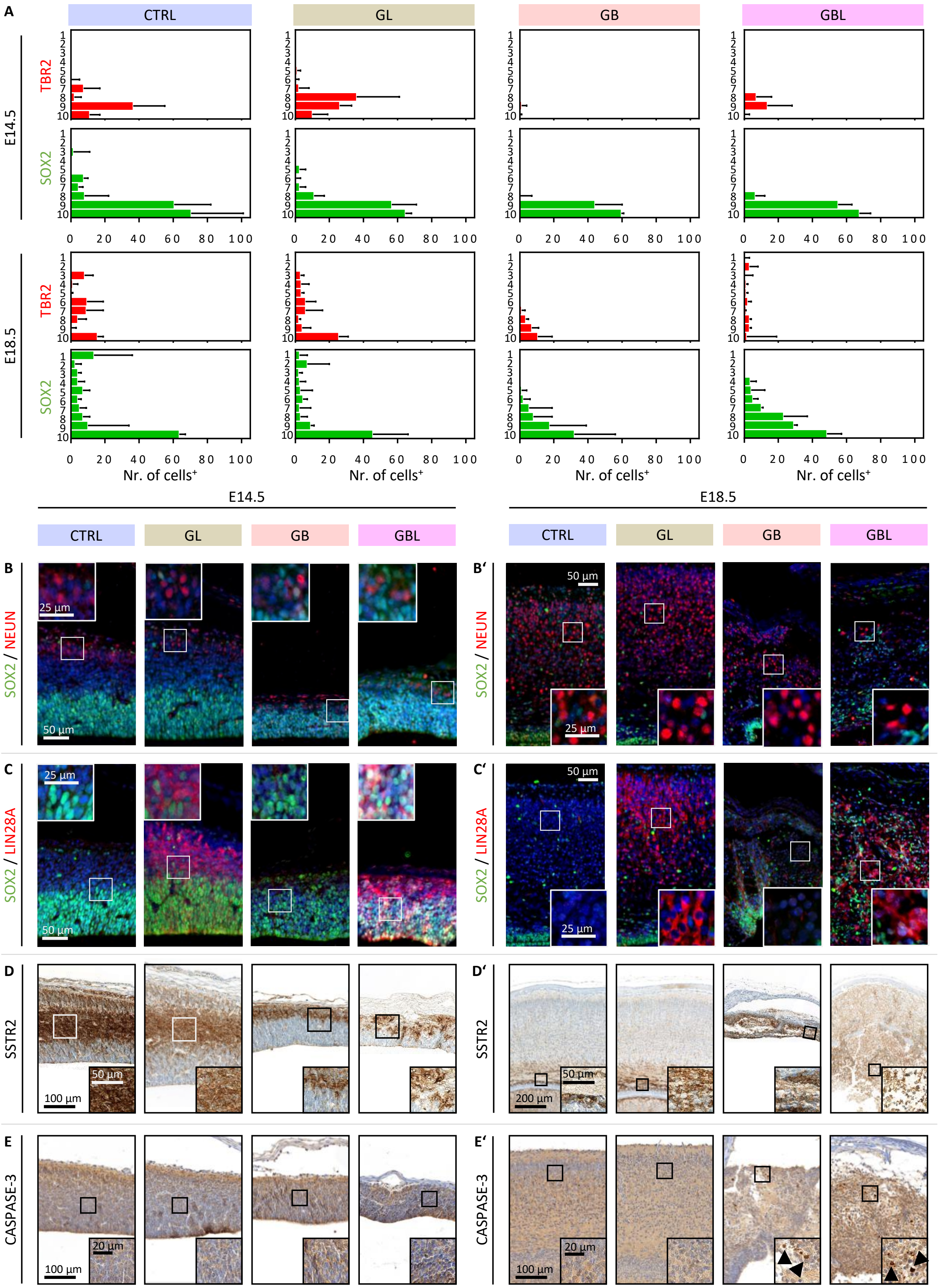

Fig. S2: Cerebral cortex marker describing cortex layering.

(A) Quantification of SOX2⁺ and TBR2⁺ cells into bins. This figure corresponds to Fig. 1E. **(B-B')** Double-Immunofluorescence of cerebral cortices at E14.5 and E18.5 in CTRL mice and the GL, GB and GBL mouse models against NEUN and SOX2, and **(C-C')** SOX2 and LIN28A. **(D-D')** Immunohistochemistry of SSTR2 and **(E-E')** CASPASE-3 at E14.5 and E18.5 in CTRL mice and the GL, GB and GBL mouse models. Arrows in **(E-E')** indicate positive cells at E18.5. For all panels: magnified region of positive area is displayed in the corner of the respective images and the scale bar for each time point. The scale applies for each time point.

Navolić et al., Fig. S3

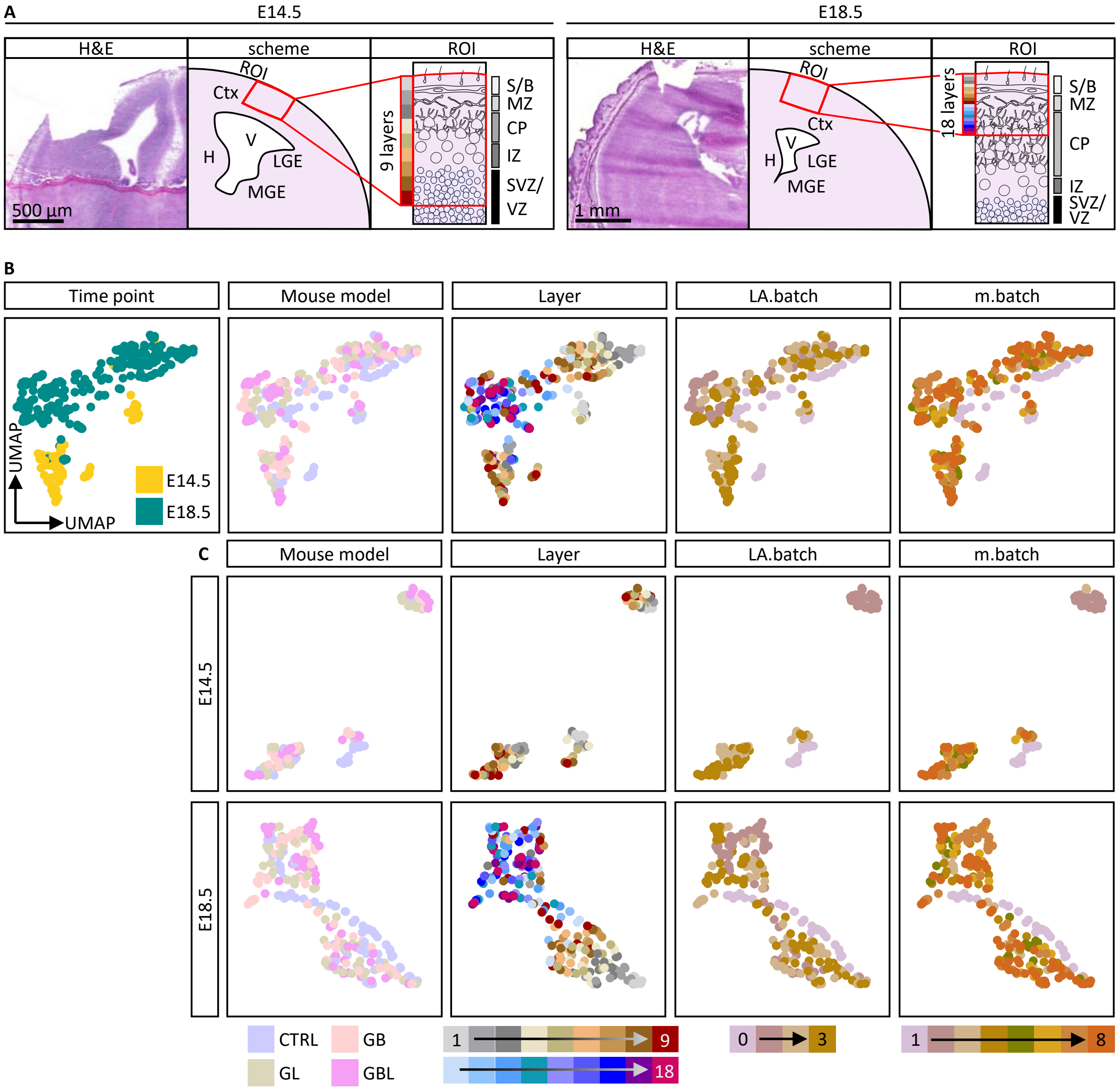

Fig. S3: Sample overview for spatial proteome analysis.

(A) Representative images of frontal H&E-stained sections at E14.5 and E18.5 after tissue ablation using NIRL. The scheme shows the region of interest (ROI) for ablation targeting the cerebral cortex (Ctx). Within the ROI, nine consecutive layers (thickness of each layer ~40 µm) were ablated from the skin into the cerebral cortex at E14.5. At E18.5 in total 18 consecutive layers were ablated. H = hippocampus, V = ventricle, MGE = medial ganglionic eminence, LGE = lateral ganglionic eminence, S/B = skin/bone, MZ = marginal zone, CP = cortical plate, IZ = intermediate zone, SVZ = subventricular zone, VZ = ventricular zone. **(B)** Uniform Manifold Approximation and Projection (UMAP) of all samples (n=367) before batch effect reduction based on proteins with 100% valid values. **(C)** UMAP displaying E14.5 and E18.5 data separately before batch effect reduction based on proteins with 100% valid values.

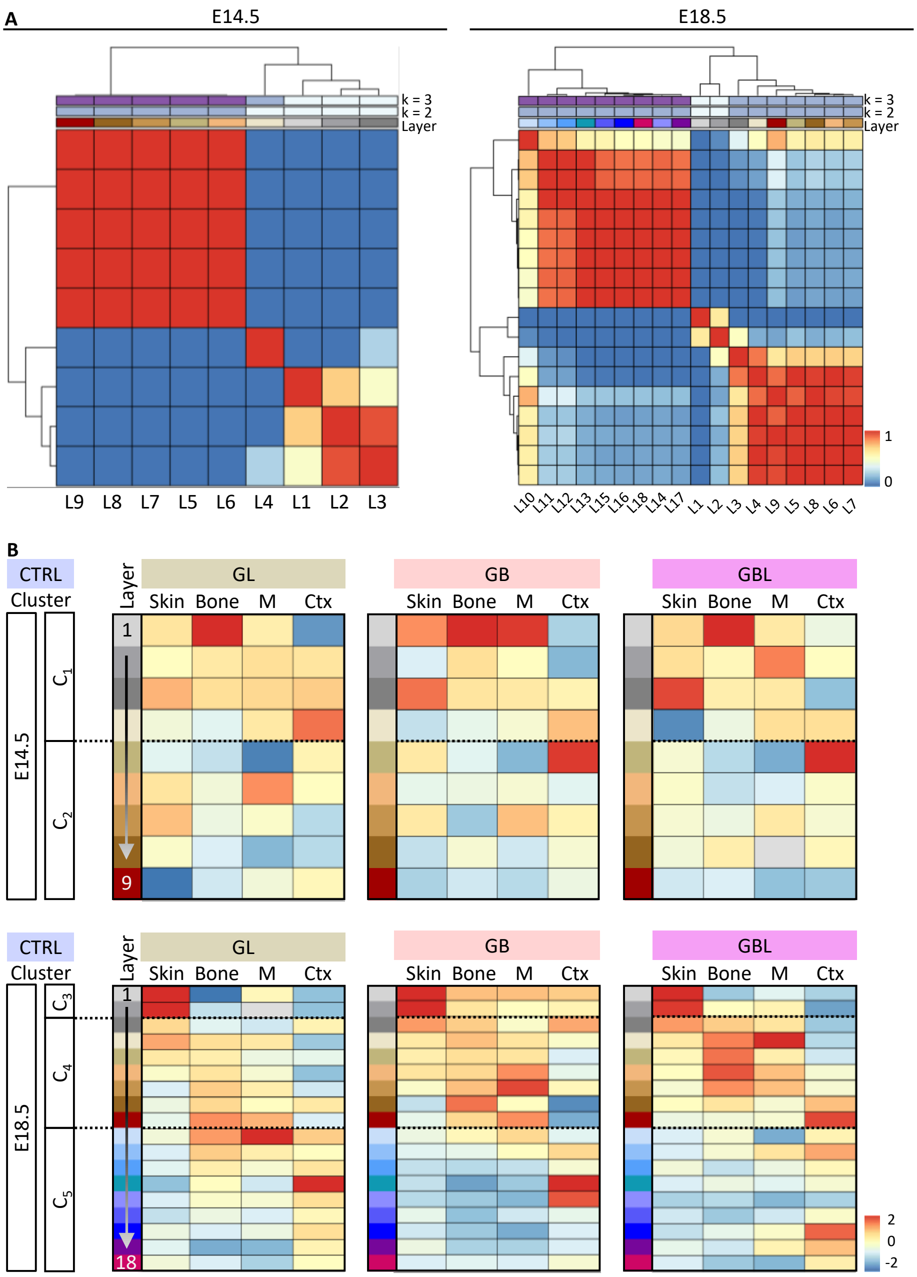

Fig. S4: Layer clustering and profiling.

(A) Heatmap of consensus clustering analysis of global proteome patterns in CTRL layers at time points E14.5 and E18.5. k = number of clusters, L = layer. **(B)** Mean column scaled abundance of marker proteins for Skin (FLG, KRT14, LORICIRIN), Bone (COL1A1, COL1A2, SERPINF1), M = Meninges (CDH11, CRABP2, TAGLN) and Ctx = cerebral cortex (TBR1, MAP2, BCL11B) in the respective mouse models GL, GB or GBL. C = cluster. C1-C5 represent clusters defined in **(A)** and are indicated with dashed lines.

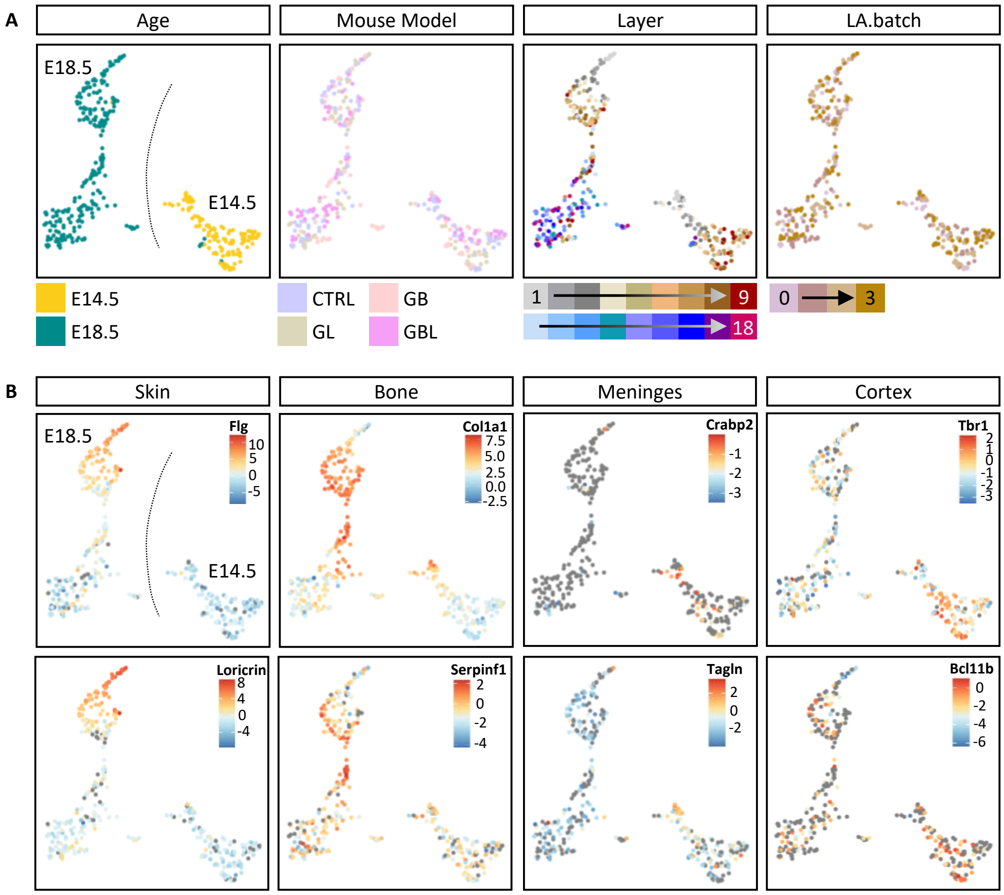

Fig. S5: Marker proteins across time points.

(A) Uniform Manifold Approximation and Projection (UMAP) of all samples of both time points E14.5 and E18.5 (n=367) after batch effect reduction based on proteins with 100% valid values. **(B)** Abundance of marker proteins for Skin (FLG, LORICIRIN), Bone (COL1A1, SERPINF1), Meninges (CRABP2, TAGLN) and Cortex (TBR1, BCL11B) mapped onto the UMAP shown in **(A)**.

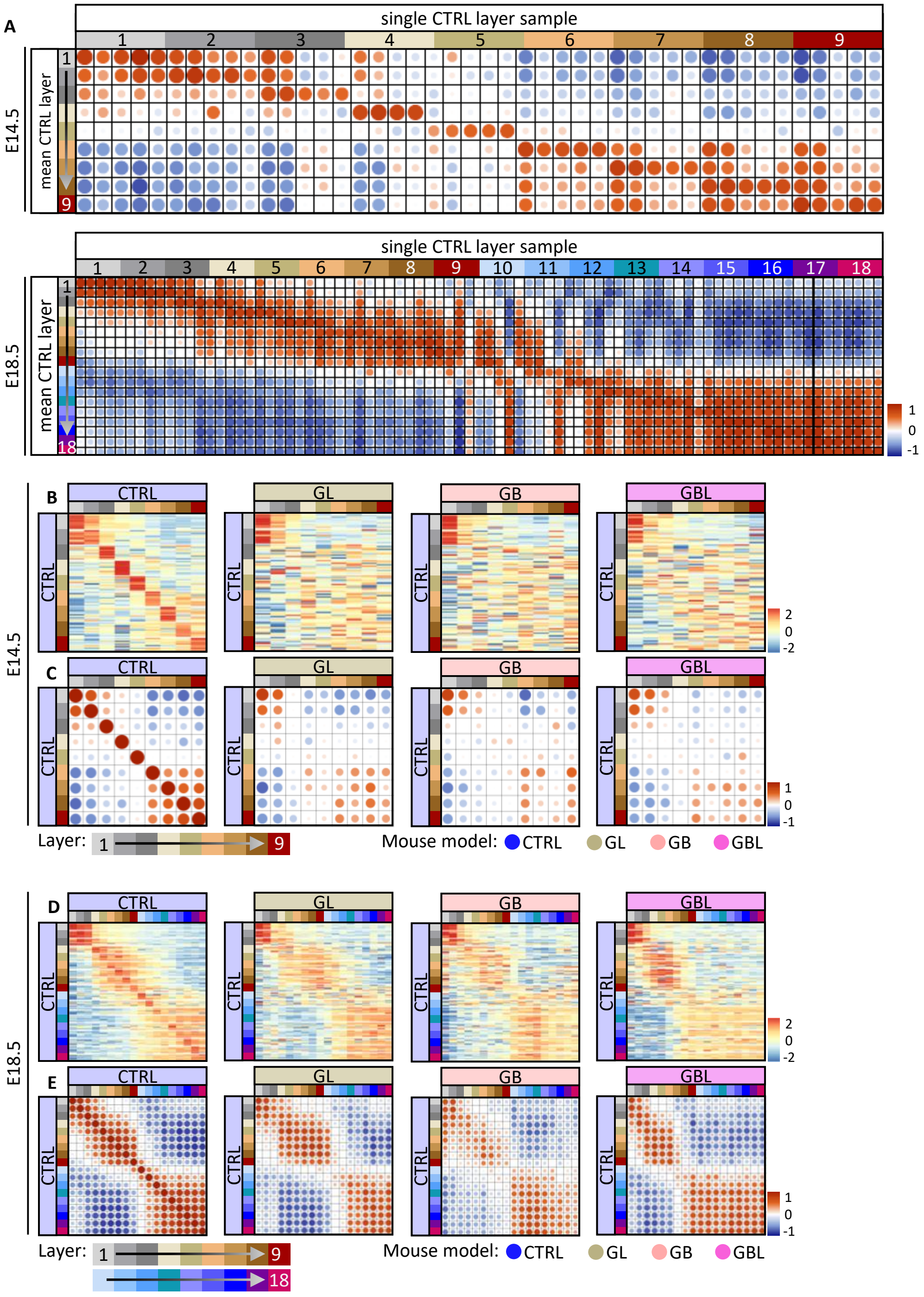

Fig. S6: Layer signatures across mouse models compared to CTRL.
(A) Correlation analysis based on the mean top 10 high abundant proteins of each CTRL layer displaying variation within the biological CTRL replicates. Protein abundances of each CTRL sample (x-axis) are correlated to the mean protein abundance of CTRL layers (y-axis). **(B, D)** Heatmap representing the top 10 high abundant proteins in each CTRL layer (y-axis) with row scaled abundances shown for CTRL, GL, GB and GBL layers (x-axis) at time point E14.5 **(B)** and E18.5 **(D)**. **(C, E)** Correlation analysis based on the top 10 uniquely high abundant proteins of each CTRL layer shown in **(B,D)**. Mean protein abundances of each CTRL, GL, GB and GBL layer (x-axis) are correlated with values of CTRL layers (y-axis).

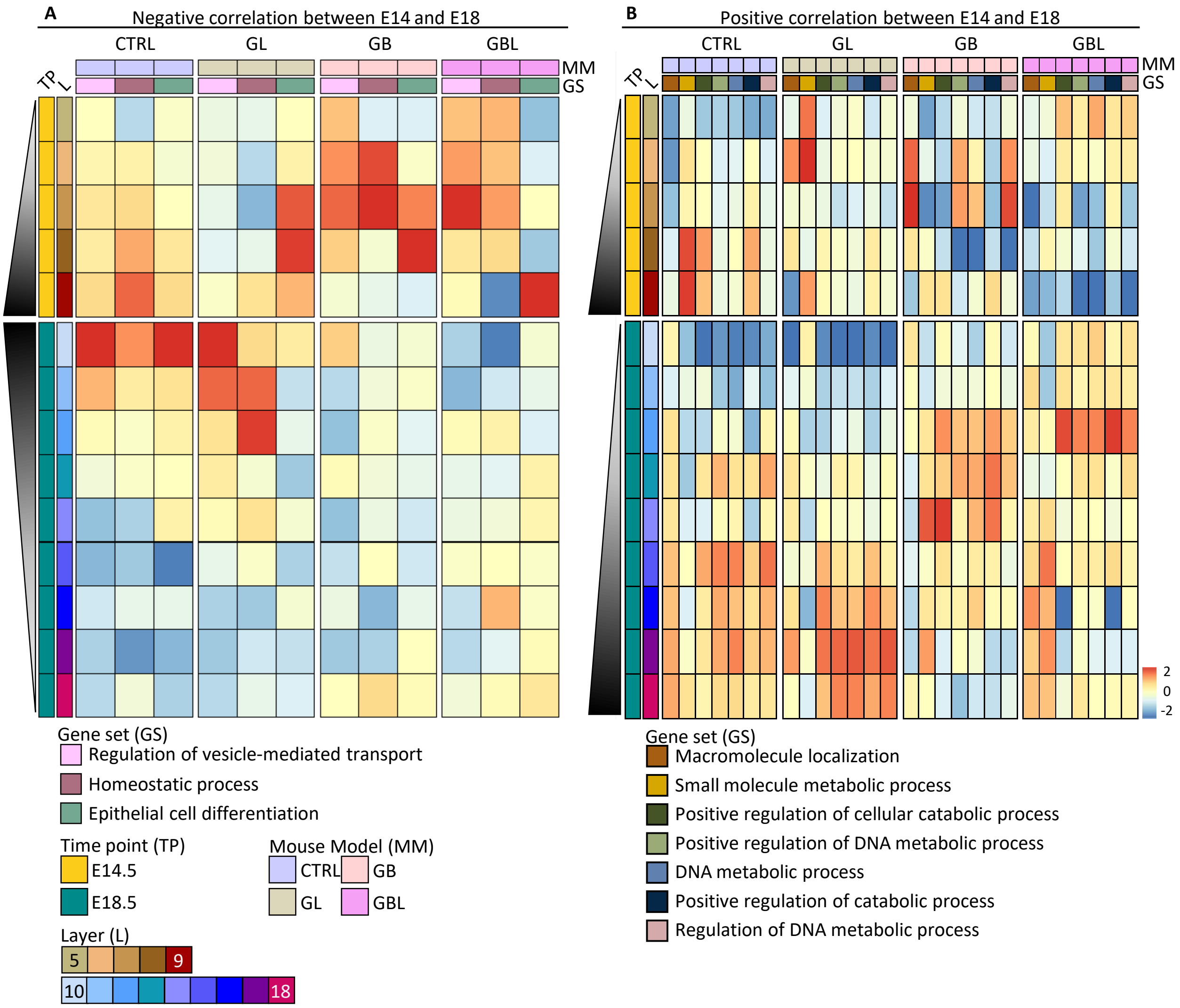

Fig. S7: Gene set representation across layers between E14 and E18.
Heatmap showing the representation of gene sets with a linear regression along the layers at both time points. **(A)** Gene sets which decrease or increase in the opposite direction (indicated by the grey bar) across the layers in the CTRL comparing both time points and showing the distribution across the mouse models. **(B)** Gene sets which increase in the same direction (indicated by the grey bar) across the layers in the CTRL comparing both time points (positive correlation) and showing the distribution across the mouse models.

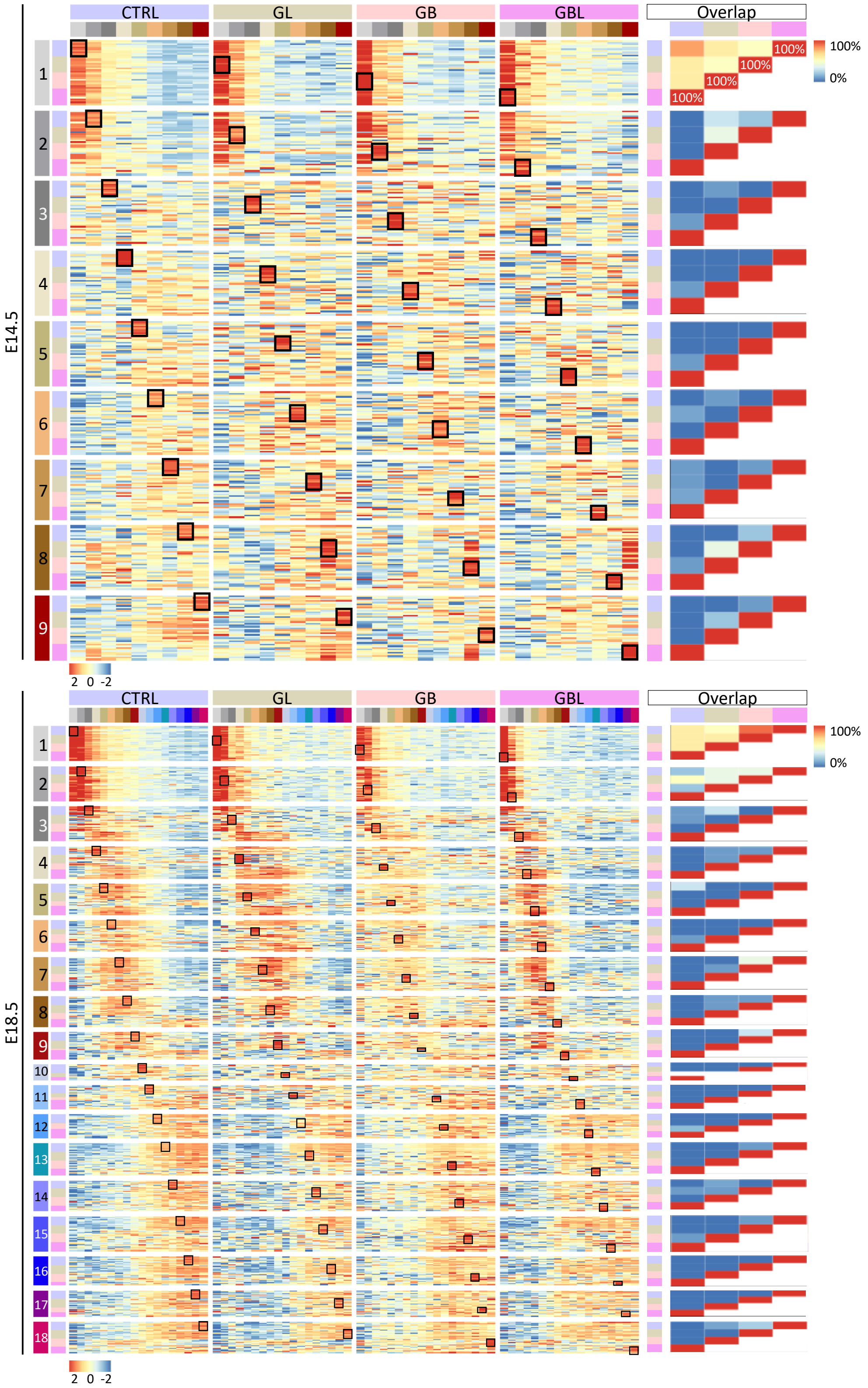

Fig. S8: Comparison of layer signatures of each mouse model.
Heatmap representing the top 10 high abundant proteins in each layer and for each mouse model (y-axis) compared in every layer and mouse model (x-axis) for both time points E14.5 and E18.5 (rows scaled). The right panel displays the percentage of overlap for the top 10 high abundant proteins for each layer comparing the genotypes. Black squares indicate the mouse model and layer-specific top 10 high abundant proteins.

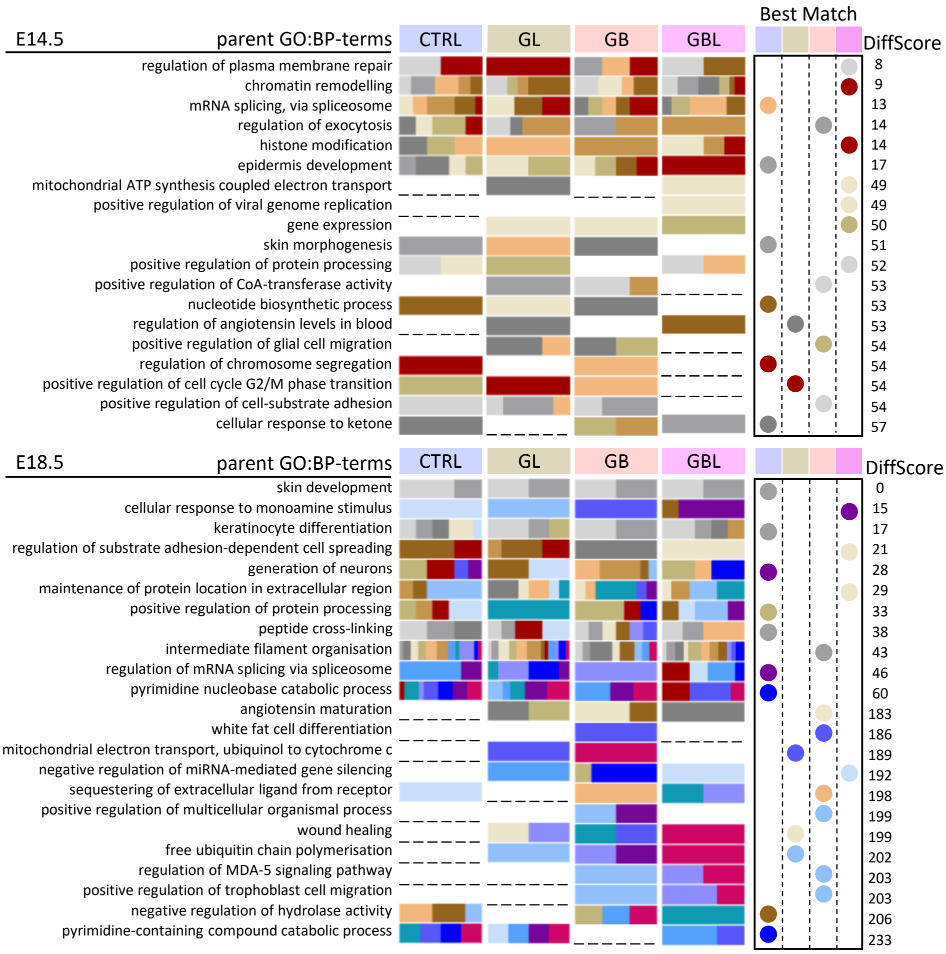

Fig. S9: Gene ontology (GO) analysis for each unique layer signature of each mouse model.
Gene ontology GO analysis for biological processes (GO:BP) based on the top high abundant proteins of each layer for each mouse model. The top 5 terms of every search were further condensed into parent GO:BP-terms followed by mouse model and layer deconvolution. Blanks mean that the parent GO:BP-term was not represented in this mouse model. The Best Match shows which mouse model and layer (colour) represents the parent GO:BP-term the best based on adjusted p-values for each GO:BP-term (Table 5). The DiffScore indicates the (spatial) distance of representative layers for each parent GO:BP-term across mouse model. In E14.5 this score can range from 0 to 90 and at E18.5 0 to 342. The higher the value, the greater the difference of the represented layers across mouse models.

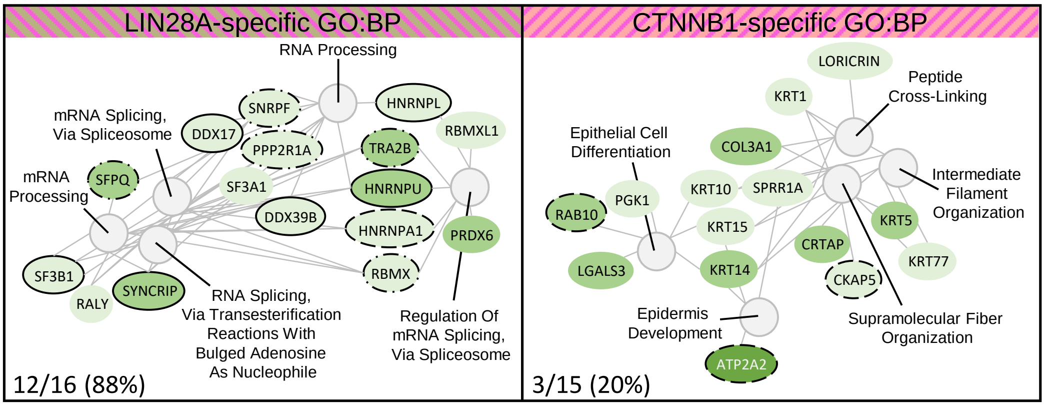

Fig. S10: LIN28A and CTNNB1 specific GO-terms.
Top 5 GO:BP-terms for LIN28A- and CTNNB1-specific protein collection. Proteins are colored based on the OmixLitMiner categories. The line types indicate previously described interaction with LIN28A. The percentage shows the proportion of potential partners to the total number of proteins for each panel (bottom corner).

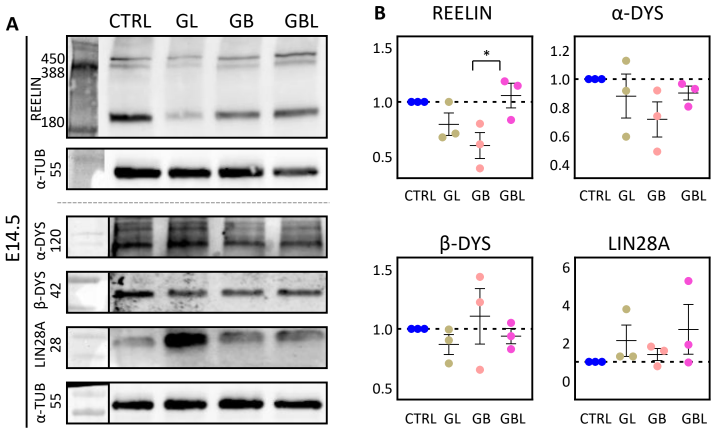

Fig. S11: Extracellular matrix component

(A) Western blot of cortical lysates stained against REELIN (450, 388, 180 kDa), α-DYS (α-DYSTROGLYCAN, 120 kDa), β-DYS (β-DYSTROGLYCAN, 42 kDa) and LIN28A (28 kDa) at E18.5. α-TUB (α-TUBULIN, 55 kDa) was used as housekeeping protein for normalization. **(B)** Respective quantification of western blot signals based on normalized values (one-way ANOVA; n >= 3, * $p < 0.05$, ** $p < 0.01$, *** $p < 0.001$; mean and SEM are shown).

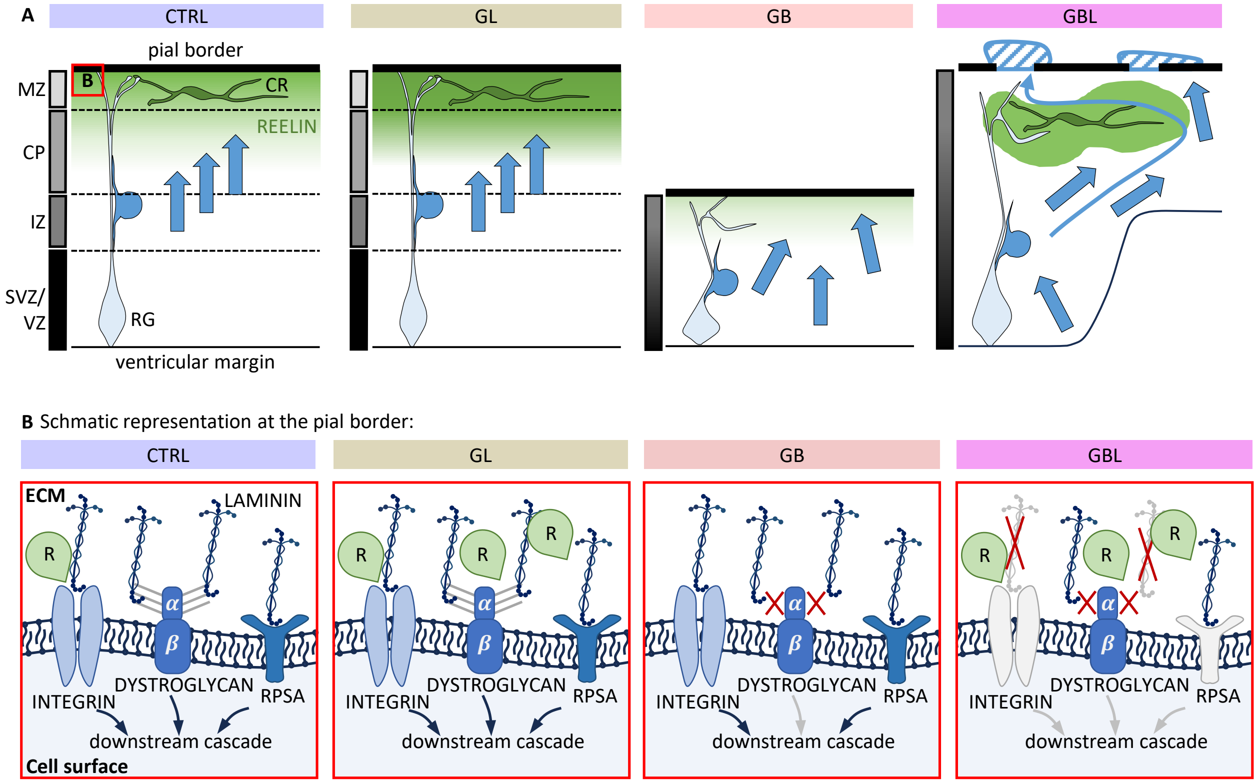

Fig. S12: Summarizing scheme of morphological and proteomic changes after LIN28A and CTNNB1 activation
(A) CTRL condition showing a representative Cajal-Retzius (CR) cell in the marginal zone (MZ) and the radial glia (RG) cell as scaffold for radial migration of neuronal cells from the ventricular zone (VZ) through the intermediate zone (IZ) to their anticipated location in the cortical plate (CP) in an insight-out manner guided by a REELIN dependent (R) gradient. Red square indicates region at the pial border described in panel **(B).** In the GL model increased REELIN expression is seen with maintained cortical migration and lamination. The GB model showed reduced REELIN expression, malformation of RGs and disturbed migration with a failure to develop proper cortical lamination. The GBL model showed various cortical thickness, disturbed lamination and porous pial border with neuronal tissue ectopically located above the pial border. CR cells were located in deeper regions of the cerebral cortex, overexpressing REELIN. **(B)** LAMININ and REELIN are components of the extracellular matrix (ECM) and are recognized by their receptors at the cell surface. Binding to the receptors INTERGIN, DYSTROGYLCAN and RPSA induces downstream signaling relevant for migration and integration of neural cells in the cortical plate. REELIN levels were increased in the GL and GBL model. Hypo-glycosylation of α- DYSTROGYLCAN was seen in the GB and GBL model, and an accumulation of disturbed spatial expression of receptor components and LAMININ was detected in the GBL model.
